# Effective rational humanization of a PASylated anti-galectin-3 Fab for the sensitive PET imaging of thyroid cancer in vivo

**DOI:** 10.1038/s41598-021-86641-0

**Published:** 2021-04-01

**Authors:** Emanuel Peplau, Francesco De Rose, Andreas Eichinger, Sybille Reder, Markus Mittelhäuser, Giorgia Scafetta, Markus Schwaiger, Wolfgang A. Weber, Armando Bartolazzi, Calogero D’Alessandria, Arne Skerra

**Affiliations:** 1 Lehrstuhl für Biologische Chemie, Technische Universität München, 85354 Freising (Weihenstephan), Germany; 2 Klinikum rechts der Isar, Nuclear Medicine Department, Technical University Munich, Ismaninger Str. 22, 81675 Munich, Germany; 3 Pathology Research Laboratory, Cancer Center Karolinska, Karolinska Hospital, 17176 Stockholm, Sweden; 4 Pathology Research Laboratory, Sant’Andrea Hospital, University Sapienza, via di Grottarossa 1035, 00189 Rome, Italy

**Keywords:** Biochemistry, Antibody fragment therapy

## Abstract

The lack of a non-invasive test for malignant thyroid nodules makes the diagnosis of thyroid cancer (TC) challenging. Human galectin-3 (hGal3) has emerged as a promising target for medical TC imaging and diagnosis because of its exclusive overexpression in malignant thyroid tissues. We previously developed a human-chimeric αhGal3 Fab fragment derived from the rat monoclonal antibody (mAb) M3/38 with optimized clearance characteristics using PASylation technology. Here, we describe the elucidation of the hGal3 epitope recognized by mAb M3/38, X-ray crystallographic analysis of its complex with the chimeric Fab and, based on the three-dimensional structure, the rational humanization of the Fab by CDR grafting. Four CDR-grafted versions were designed using structurally most closely related fully human immunoglobulin V_H_/V_L_ regions of which one—employing the acceptor framework regions of the HIV-1 neutralizing human antibody m66—showed the highest antigen affinity. By introducing two additional back-mutations to the rodent donor sequence, an affinity toward hGal3 indistinguishable from the chimeric Fab was achieved (K_D_ = 0.34 ± 0.02 nM in SPR). The PASylated humanized Fab was site-specifically labelled with the fluorescent dye Cy7 and applied for the immuno-histochemical staining of human tissue sections representative for different TCs. The same protein was conjugated with the metal chelator Dfo, followed by radiolabelling with ^89^Zr(IV). The resulting protein tracer allowed the highly sensitive and specific PET/CT imaging of orthotopic tumors in mice, which was confirmed by quantitative analysis of radiotracer accumulation. Thus, the PASylated humanized αhGal3 Fab offers clinical potential for the diagnostic imaging of TC.

## Introduction

Thyroid cancer (TC) generally occurs in the form of nodules in the thyroid parenchyma or of small sclerotic areas with irregular borders. The prevalence of thyroid nodules is very high in geographic regions with iodine deficiency and may reach up to 68% in the adult population according to recent studies^1,2^. However, as the overwhelming majority of thyroid nodules is benign, the challenge lies in their distinction from malignant lesions^3,4^. Currently, thyroid ultrasound (US) and fine needle aspiration biopsy (FNA) are the standard tools to diagnose TC. However, in particular differentiated TC (DTC) with follicular structure expressing thyroglobulin is hard to distinguish from benign nodules preoperatively, due to the common cyto-architectural features. Apart from better diagnostic tools in general, there is also a medical need for improved therapies of poorly differentiated TC (PDTC) and anaplastic TC (ATC). These two forms of TC are frequently fatal, exhibit rapid growth with metastases in up to 75% of the patients, and they are often recognized and diagnosed late at advanced stages^5^.

Both thyroid US imaging and FNA have contributed to consistently improving the diagnosis of thyroid nodules, but both do not easily allow preoperative discrimination between benign and malignant lesions. As a consequence, the surgical overtreatment of benign thyroid nodules is common^6,7^ because so far only histology can reliably differentiate between follicular TC and benign thyroid nodules. Thus, the preoperative characterization of thyroid nodules is clinically highly relevant because it would facilitate selection of patients to be referred to surgery, thus contributing to better TC follow-up as well as treatment optimization. In this regard, the overexpressed β-galactoside-binding lectin galectin 3 (Gal3) has shown promise as a molecular target for the diagnosis of TC, as this phenotypic feature is absent in normal thyroid cells^8^.

To exploit Gal3 overexpression for tumor imaging via positron emission tomography (PET) we recently developed a ^89^Zr-labeled chimeric Fab-fragment derived from a well characterized rat monoclonal antibody (mAb M3/38) which demonstrated high imaging contrast in mice^9^. In accordance with earlier in vivo imaging studies of other tumor targets^10,11^, a Fab version with moderately prolonged plasma half-life utilizing PASylation technology turned out to result in excellent and highly specific uptake in orthotopic thyroid tumor models.

With the goal of clinical translation, further aspects beyond good tumor penetration and rapid clearance have to be considered. While the preparation of a chimeric Fab—here comprising variable domains from rat combined with human constant domains—considerably reduces the immunogenicity of the protein reagent compared with a natural rodent antibody (also owing to the absence of the Fc portion), there are still reported cases of an anti-drug antibody (ADA) response directed against the non-human V-regions^12^. A solution to this problem is the humanization of the Fab via grafting of the complementarity determining regions (CDRs) from rat onto a human immunoglobulin (Ig) framework as initially demonstrated more than 30 years ago^13,14^. In fact, since the first market approval of trastuzumab for the treatment of HER2-positive metastasizing breast cancer, CDR grafting has become a generally accepted strategy for biopharmaceutical mAb development up to now^15,16^.

However, a successful humanization campaign depends on a high structural similarity between donor and acceptor Ig frameworks since even minute conformational changes in the paratope upon CDR transplantation may critically affect the antigen-binding activity^17^. Therefore, we have elucidated the crystal structure of the chimeric Fab in complex with its antigen as a prerequisite for the informed choice of a suitable human Ig scaffold. This enabled the functional CDR grafting via rational protein design, resulting in a humanized αGal3 Fab that allowed sensitive PET imaging of orthotopic human thyroid tumors in mice.

## Results

### Gal3 epitope determination

**Figure 1:**
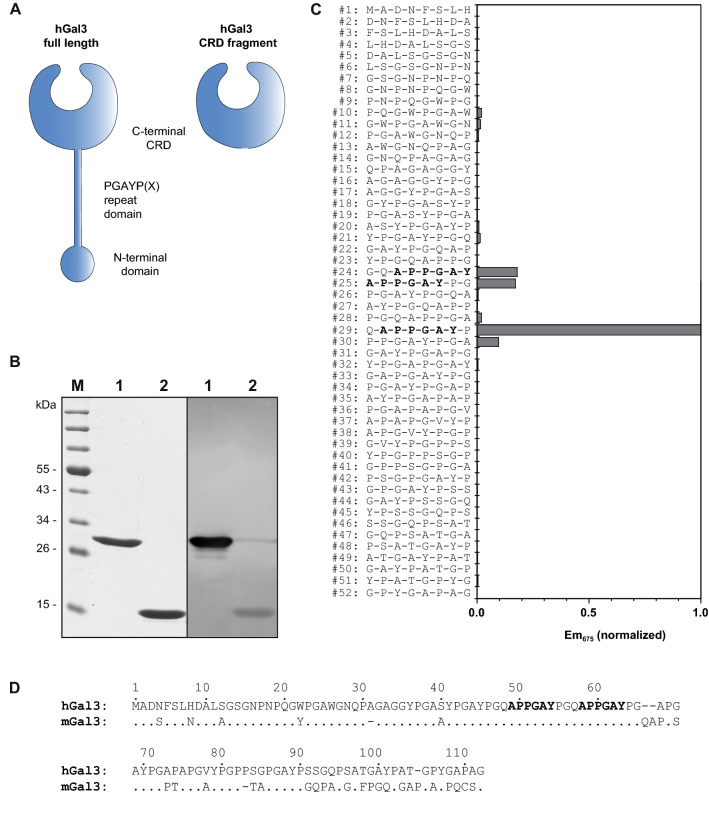
Elucidation of the hGal3 epitope recognized by the M3/38 Fab. (**A**) Schematic representation of the full-length hGal3 (27 kDa) and its truncated CRD (residues 113–252; 14 kDa) which were investigated here. (**B**) Western-blot analysis of the full length recombinant hGal3 and its CRD with the chimeric αhGal3-Fab-PAS200-Cy5.5. Lanes: M, molecular size standard; 1, full length hGal3; 2, hGal3 CRD. Left: coomassie-stained SDS-PAGE. Right: western-blot probed with the fluorescent chimeric αhGal3-Fab-PAS200-Cy5.5. (**C**) Linear epitope scan of the ND of hGal3 using the SPOT technique and the chimeric αhGal3-Fab-PAS200-Cy5.5 for direct detection by fluorescence at 675 nm (normalized to 1). The 8mer peptides cover the sequence of the ND of hGal3 as shown in (**D**) with a two-residue offset. (**D**) Amino acid sequence alignment between the NDs of human and murine Gal3 (sequence numbers are indicated for the human protein). The epitope of the αhGal3-Fab seen in (**C**) is highlighted in bold. Graphics were prepared using Quant version 12.2 (https://totallab.com) and Origin(Pro) version 2017 (https://www.originlab.com).

Human Gal3 (hGal3) comprises a C-terminal carbohydrate recognition domain (CRD) and a characteristic flexible N-terminal domain (ND) of 113 residues with several 9-amino acid repeats: Pro-Gly-Ala-Tyr-Pro-Gly-Xaa-Xaa-Xaa^18^ (Fig. 1). Due to the inherent flexibility of the ND, hGal3 represents a challenging target for structural analysis. In fact, the crystal structure of full length Gal3 is unknown to date, which prompted us to narrow down the epitope that is recognized by the mAb M3/38 or its chimeric recombinant Fab^9^. On a western blot of both the full length recombinant hGal3 and a truncated version lacking the ND only the complete antigen led to a signal with the fluorescence-labelled chimeric αhGal3-Fab-PAS200-Cy5.5^9^ (Fig. 1B), indicating that a linear epitope within the ND is recognized.

Thus, the SPOT technique^19^ was applied to identify the epitope using an array of consecutive synthetic hGal3 peptides that were immobilized on a hydrophilic membrane. To this end, a set of 45 8mer peptides, each shifted by 2 residues and covering the sequence of the full ND (residues 1–112), was synthesized. Incubation with the Cy5.5-labelled chimeric αhGal3-Fab-PAS200 led to prominent fluorescent binding signals for three peptide spots, nos. 24, 25 and 29 (Fig. 1C), whereby the first two signals correspond to peptides with overlapping sequences. The common minimal sequence motif of all three spots was: Ala-Pro-Pro-Gly-Ala-Tyr. Of note, this linear epitope occurs in two of the 9-residue repeats within the hGal3 ND mentioned above.

### Crystallographic analysis of the αhGal3-Fab·peptide complex

The recombinant chimeric Fab was functionally produced in *E. coli* and purified to homogeneity as previously described^9^, then mixed with the N-terminally acetylated synthetic peptide Gln-Ala-Pro-Pro-Gly-Ala-Tyr-Pro-Gly. The protein crystallized in the presence of 25% (w/v) PEG4000, 0.1 M HEPES/NaOH pH 7.0 in the space group C2 with one Fab·peptide complex in the asymmetric unit. A synchrotron X-ray diffraction data set at 1.9 Å resolution was collected and the structure of the complex was solved by molecular replacement (see “Materials and methods”). Residues Asp(L1)–Cys(L214) of the light chain and Gln(H1)–Cys(H230) of the heavy chain (residue numbering according to Kabat^20^) as well as the complexed 9mer peptide were defined in the electron density map, with weaker density for residues Cys(L214), Gly(H42), Lys(H43) and Cys(H230). In total 5 peptide bonds with *cis*-configuration were found at typical positions of the Ig chains: Thr(L7)–Pro(L8), Phe(L94)–Pro(L95), Tyr(L140)–Pro(L141), Phe(H148)–Pro(H149) and Glu(H150)–Pro(H151). Also, the five conserved disulfide bridges, including the one linking the light and heavy chains, Cys(L214)–Cys(H230) (with partial definition), were visible. Residues Val(L51) and Ser(H229) exhibit outlier ϕ/φ angles in the Ramachandran plot (Table 1) but are well defined in the electron density.

**Table 1:** Crystallographic analysis and refinement statistics.

	Fab M3/38·hGal3 peptide
*Crystal Data:*	
Space group	C2
**Unit cell dimensions**	
a, b, c [Å]	94.1, 61.3, 80.9
α, β, γ [°]	90.0, 103.4, 90.0
Molecules per asym. unit	1 complex
**Data collection**	
Wavelength [Å]	0.91840
Resolution range [Å]^a^	78.72–1.90 (2.00–1.90)
I/σ[I]^a^	5.7 (1.8)
R_merge_ [%]^a, b^	8.8 (41.1)
Unique reflections	34,354
Multiplicity^a^	5.7 (5.9)
Completeness^a^	97.0 (97.9)
**Refinement**	
R_cryst_/R_free_^c^	20.3/24.3
Protein atoms	3367
Peptide atoms	64
Solvent atoms	201
Average B-factor [Å^2^]	
Protein	27.3
Peptide	35.9
Water	32.3
**Geometry**	
R.m.s.d. bond lengths, angles [Å, °]	0.007, 1.519
Ramachandran analysis^d^: core, allowed, generously allowed, disallowed [%]	89.0, 9.9, 0.8, 0.3

^a^Values in parentheses are for the highest resolution shell.

^b^$${\text{R}}_{{{\text{merge}}}} = \sum\nolimits_{{\text{h}}} {\sum\nolimits_{{\text{i}}} {\left| {l_{{\text{i}}} ({\text{h}}) - \left\langle {l({\text{h}})} \right\rangle } \right|} } /\sum\nolimits_{{\text{h}}} {\sum\nolimits_{{\text{i}}} {l_{{\text{i}}} ({\text{h}})\quad } } {\text{R}}_{{{\text{cryst}}}} = \sum\nolimits_{{\text{h}}} {\left| {\left| {{\text{F}}_{{\text{o}}} {\text{(h)}}} \right| - \left| {{\text{F}}_{{\text{c}}} {\text{(h)}}} \right|} \right|} /\sum\nolimits_{{\text{h}}} {\left| {{\text{F}}_{{\text{o}}} {\text{(h)}}} \right|} .$$

^c^R_free_ is R_cryst_ with 5% of the reflections that were randomly selected and excluded from refinement^79^.

^d^Calculated with PROCHECK.

**Figure 2:**
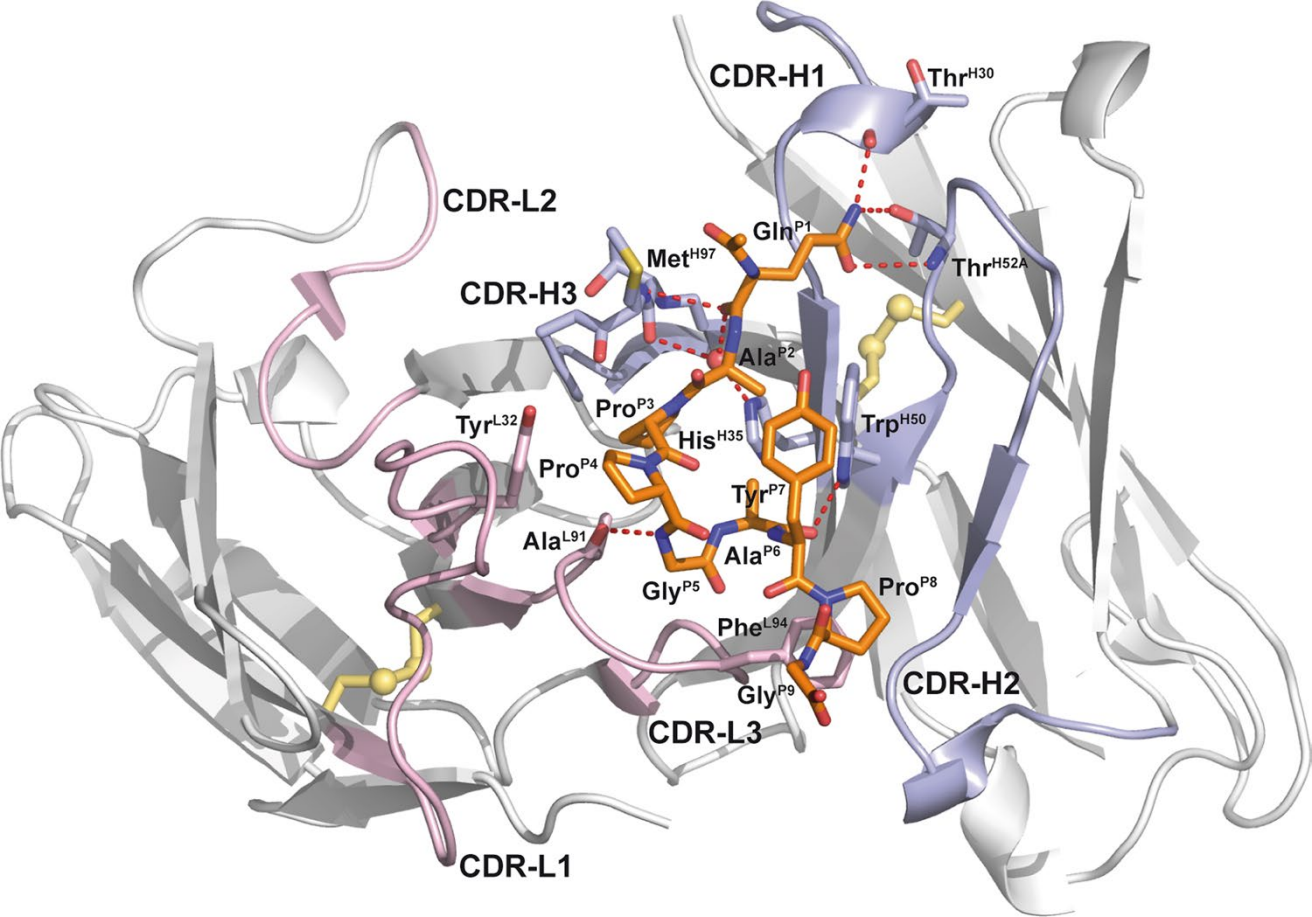
Crystal structure of the chimeric αhGal3-Fab fragment (Fv portion) in complex with the hGal3 epitope peptide, Ac-Gln-Ala-Pro-Pro-Gly-Ala-Tyr-Pro-Gly. The (N-acetylated) hGal3 peptide (residues P1-P9, orange) is bound in a cleft between both variable domains. The secondary structure elements are representated as cartoons and colored in light gray while disulfide bonds are shown yellow in ball-and-stick representation; the CDRs of the light and heavy chains are colored in light pink and light blue, respectively. The mAb residues that are engaged in aromatic contacts or form hydrogen bonds (red dotted lines) to the hGal3 peptide, together with the two residues that form bridged hydrogen bonds through a bound water molecule (red sphere), are depicted as sticks. Those residues that make direct hydrogen bonds to the epitope peptide are labeled (see Table 2). Graphics were prepared using PyMOL version 1.30 (https://www.schrodinger.com).

The three-dimensional structure of the rat/human chimeric Fab exhibits the typical fold of a Fab fragment as known from human and rodent Igs (Fig. 2). The paratope comprises a shallow cleft between the V_H_ and V_L_ domains about 12 Å by 18 Å wide and 9 Å deep. Within this cleft the 9-residue hGal3 epitope peptide is bound primarily by CDR-H3 and CDR-L3. The peptide shows an elongated, curved backbone conformation including a short 3_10_-helix formed by residues Pro(P4) to Ala(P6). A PISA analysis^21^ revealed that 61.4% of the total solvent-accessible surface area of the peptide (647 of 1053 Å^2^) is buried in the binding site. Altogether 26 residues of the Fab form van der Waals contacts to the bound peptide with a contact surface greater than 1 Å^2^, 17 arising from the heavy chain and 9 from the light chain; of these, 10 are hydrophobic and 15 polar (Table 2). Furthermore, 4 residues in the heavy chain and one in the light chain form hydrogen bonds to the peptide: Ala(L91), Thr(H30), Trp(H50), Thr(H52A) and Met(H97). Apart from Trp(H47), all contacting residues are located within CDRs.

**Table 2:** Residues of the Fab M3/38 that form van der Waals contacts with the hGal3 peptide in the complex.

Residue	BSA [Å^2^]	Interaction	Residue	BSA [Å^2^]	Interaction
Thr(H30)	4.30	HB	His(L27D)	22.26	
Asp(H31)	13.70		Asp(L28)	5.77	
Tyr(H32)	2.93		Tyr(L32)	27.40	
Ala(H33)	20.88		Trp(L89)	2.50	
His(H35)	18.81	(HB)^a^	Ala(L91)	27.81	HB
Trp(H47)	19.95		Thr(L92)	8.77	
Trp(H50)	59.51	HB	His(L93)	2.20	
Asn(H52)	19.39		Phe(L94)	85.58	
Thr(H52A)	2.34	HB	Leu(L96)	30.12	
Tyr(H53)	3.85				
Ile(H58)	50.33				
Tyr(H59)	1.13				
Lys(H64)	2.37				
Gly(H95)	1.50				
Thr(H96)	16.87	(HB)^a^			
Met(H97)	60.21	HB			
Ala(H99)	21.93				

Buried surface areas (BSA) > 1 Å^2^ are reported for each of the contacting amino acids and hydrogen bonds (HB) are indicated.

^a^Indirect H-bond (mediated by a water molecule).

There are also some other relevant features of this antibody-antigen complex: Gln(P1) forms with its side chain carboxamide group one and two hydrogen bonds, respectively, to residues Thr(H30) and Thr(H52A) as well as with its main chain carbonyl oxygen hydrogen bonds to Met(H97) and to the only water molecule that is buried in the cleft (see Fig. 2). The side chain of Trp(H50) in CDR-H2 forms a hydrogen bond to peptide residue Ala(P6) and also an edge-on contact to the aromatic side chain of Tyr(P7). In a similar way, the peptide residue Pro(P4) contacts with its Cγ-atom the aromatic plane of the Tyr(L32) side chain in CDR-L1. The aliphatic ring of Pro(P8), on the other hand, stacks against the side chain of Phe(L94) in CDR-L3.

### Humanization of the mAb M3/38V-regions

Basis of our humanization approach was a structural alignment of the X-ray structure determined for the chimeric αhGal3-Fab against the AbDB, a specialized collection of pre-numbered non-redundant antibody Fv portions of antibodies with known three-dimensional structures^22^. For this alignment only a subset of those 695 Fv structures with a reported human origin was chosen. The Cα-atom superposition was conducted with PDBeFold, which rates each match based on structural similarity and paired amino acid sequence length^23^. The first 20 hits resulting from this search showed a narrow distribution for the ranking factor Q, between 0.89 and 0.85 (Table 3).

**Table 3:** List of human Fv fragments from the AbDB^22^ with high structural similarity to the V-regions of Fab M3/38 based on an alignment with PDBeFold^21^.

#	Q	R.M.S.D	N_Align_	N_Residue_	Seq. identity [%]	Source	PDB ID
1	0.85	0.89	220	229	53	Humanized	1L7I
**2**	**0.85**	**1.00**	**222**	**230**	**54**	**Human**	**5ILC**
**3**	**0.84**	**1.01**	**222**	**232**	**50**	**Human**	**5I8C**
**4**	**0.84**	**0.99**	**218**	**225**	**54**	**Human**	**4KQ3**
5	0.83	1.07	222	231	57	unpublished	3NCJ
**6**	**0.83**	**1.03**	**220**	**229**	**54**	**Human**	**3KYM**
7	0.83	1.07	218	223	52	Humanized	5TDO
8	0.83	1.08	222	231	57	unpublished	3NAA
**9**	**0.83**	**1.03**	**220**	**229**	**57**	**Human**	**5V7R**
10	0.82	1.10	222	231	56	unpublished	3NAB
**11**	**0.82**	**1.09**	**222**	**232**	**54**	**Human**	**4NRY**
12	0.82	1.17	221	226	53	Humanized	1T3F
**13**	**0.82**	**1.07**	**223**	**235**	**60**	**Human**	**5ILL**
14	0.82	1.13	223	232	54	Human	4NRY
**15**	**0.82**	**1.07**	**217**	**223**	**51**	**Human**	**5TDN**
**16**	**0.82**	**1.18**	**222**	**228**	**57**	**Human**	**5IL6**
**17**	**0.82**	**1.07**	**222**	**234**	**56**	**Human**	**5IT2**
18	0.82	1.12	221	230	50	Humanized	1AD0
**19**	**0.82**	**1.15**	**221**	**229**	**53**	**Human**	**4LLU**
**20**	**0.81**	**1.22**	**221**	**226**	**48**	**Human**	**2JIX**

This analysis was performed with a coordinate set at a preliminary stage of refinement (R_cryst_ = 20.7%/R_free_ = 25.1%).

*Q* mean square deviation weighted by the length of the alignment, N_Align_; *N*_*Residue*_ total number of amino acid residues in the sequence; *R.M.S.D*. root mean square deviation.

**Figure 3:**
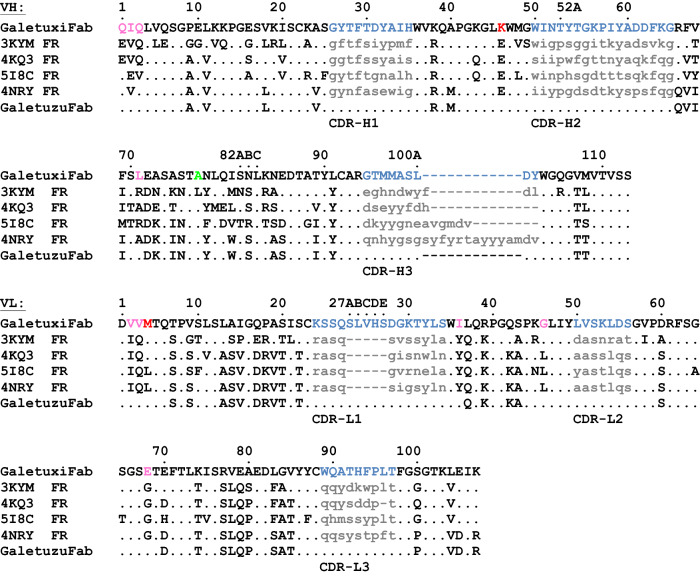
Amino acid sequence alignment of the V-regions cloned from the M3/38 hybridoma in form of the chimeric αhGal3-Fab (GaletuxiFab) with suitable human template sequences for CDR grafting as well as the finally humanized version, GaletuzuFab. Human V-region templates considered in this study were from a mAb targeting the surface glycoprotein LINGO-1 (PDB ID: 3KYM), an anti-IL-17A antibody (PDB ID 4KQ3), the HIV-1 neutralizing Fab VRC34.01 (PDB ID: 5I8C) and from the HIV-1 neutralizing antibody m66 (PDB ID: 4NRY), which appeared to offer the most promising acceptor framework to yield GaletuzuFab. Sequences are numbered according to Kabat^20^. CDRs of the donor antibody are highlighted in blue whereas those of the acceptor antibodies are shown in lower case letters. The definition of CDR-L1 also includes positions L26–L30 as proposed by Chothia et al.^31^. Initially introduced back-mutations for CDR grafting are highlighted in pink (with an additional position for 3KYM in green), second generation back-mutations are colored red.

A following literature search revealed an unclear origin or not fully human nature for 10 of these 20 candidates, as some of them were already result of a previous humanization campaign, for example. The remaining 10 original human Fv structures were further assessed for their potential as an acceptor scaffold for CDR grafting. This endeavour was guided by the plausible assumption that a close structural similarity between animal donor and human acceptor framework regions will most likely retain the functional CDR conformations after grafting^17^. First, a rational analysis of the Fv structures was performed using computer graphics. A Cα alignment of each potential acceptor framework (residues L1–23, L35–49, L57–88, L98–107, H1–25, H36–49, H66–94, H103–111; numbering according to Kabat^20^) with the crystal structure of the chimeric αhGal3-Fab described above allowed us to spot critical regions that might influence the conformations of the grafted CDRs (i.e., residues L24–34, L50–56, L89–97, H26–35, H50–65, H95–102; cf. Fig. 3). From this analysis it appeared that four Fv structures derived from the PDB showed the least conflicts: PDB ID 3KYM, a mAb targeting the surface glycoprotein LINGO-1^24^, PBD ID 4KQ3, an anti-IL-17A antibody^25^, PDB ID 5I8C, the HIV-1 neutralizing Fab VRC34.01^26^, and PDB ID 4NRY, the HIV-1 neutralizing antibody m66^27^.

**Figure 4:**
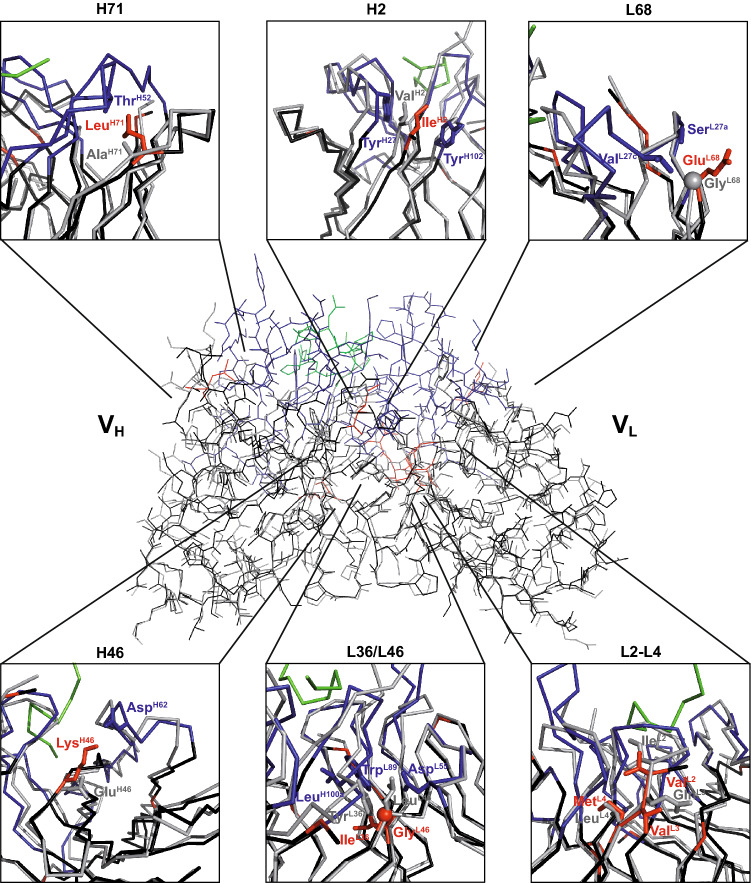
Structural superposition of the chimeric αhGal3-Fab with the crystal structure of mAb m66 (PDB ID: 4NRY) via Cα positions of the framework regions in both variable domains. The framework residues of the αhGal3-Fab are shown in black, V_H_ CDRs in dark blue, V_L_ CDRs in light blue, the hGal3 epitope peptide in green, the m66 framework residues in grey. Side chains of the backmutated framework residues are highlighted as sticks (αhGal3-Fab: red; m66: grey; structurally interfering residues: blue, see text). Graphics were prepared using PyMOL version 1.30 (https://www.schrodinger.com).

Structural inspection indicated that despite the high similarity between the rat framework amino acid sequences of mAb M3/38 and each of the four human target Fv portions (Fig. 3), residues Leu(H71), Ala(H78) (only in 3KYM), Ile(L36), Gly(L46) and Glu(L68) within the so-called vernier region^28^ of the rodent αhGal3-Fab had to be reconstituted in the human acceptor sequences (Fig. 4). Furthermore, it was known from the cloning of the V-regions of mAb M3/38 from the hybridoma cell line that the N-termini of both Ig chains influence the antigen affinity^9^; therefore, all human acceptor sequences were adapted to the rat Ig via mutation (to the extent necessary, see Fig. 3) to Glu(H1), Ile(H2), Glu(H3) and Val(L2), Val(L3).

In the human V_H_ sequence the positions H2 and H71 were substituted by the corresponding residues from the rat mAb according to the following rationale. Residue H2 is situated underneath the CDR-H3, which forms a bent hairpin loop in the crystal structure of the chimeric αhGal-Fab (see Figs. 2, 4). Especially the interactions with the residues at the base of this loop are crucial for the orientation of CDR-H3 and thereby relevant for antigen binding^29^. Residue H71 is known to influence the conformations of both CDR-H2 and CDR-H3; depending on the size of its side chain, CDR-H2 gets shifted relative to CDR-H3^30^.

In the human V_L_ sequence altogether five positions were substituted: L2, L3, L36, L46 and L68. The effect of the N-terminal residues on the antigen affinity was already demonstrated during the cloning of the functional chimeric αhGal3-Fab from the hybridoma^9^. In fact, L2 has been described as a critical residue that forms a platform for CDR-L1^31^. Positions L36 and L46 represent a pair of back-mutations in the vernier zone of the V_L_ domain. Both are residues at the hydrophobic V_L_/V_H_ interface. The size of these side chains can influence the angle between the V_H_ and V_L_ domains, which would change the relative positions of the CDRs from heavy and light chains and thereby the shape of the paratope^32^. Finally, residue L68 is located at the tip of a hairpin loop on the backside of CDR-L1, such that a size difference between the human and rodent side chains would provoke a shift of this CDR.

**Figure 5:**
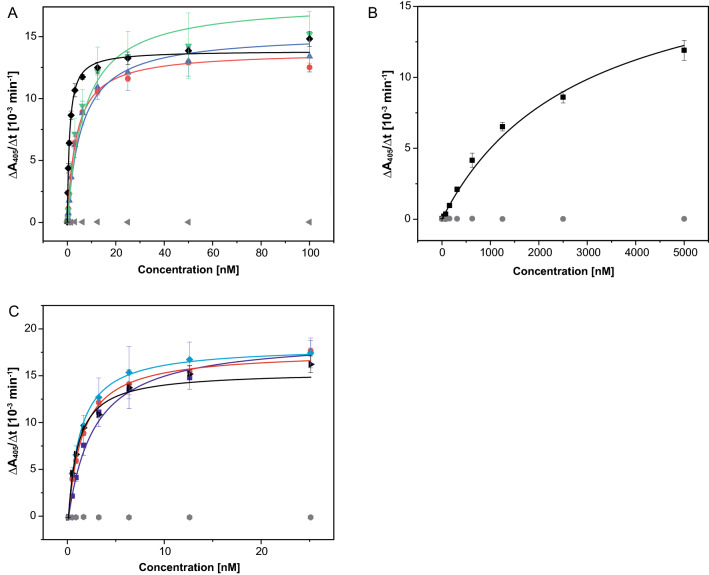
Investigation of the antigen-binding activity of different humanized Fab versions by ELISA. Recombinant hGal3 was immobilized on a microtiter plate, then a dilution series of the different Fabs was applied and, after washing, bound Fab was detected with a goat anti-human-kappa-light-chain-IgG alkaline phosphatase conjugate. (**A**) Chimeric αhGal3-Fab (black; K_D_ = 1.3 ± 0.2 nM) and the initial CDR-grafted versions "4NRY" (red; K_D_ = 3.6 ± 0.1 nM), "4KQ3" (blue; K_D_ = 5.9 ± 1.9 nM) and "5I8C" (green; K_D_ = 6.9 ± 1.3 nM). (**B**) The same experiment shown for the humanized Fab version "3KYM" (black; K_D_ = 2.8 ± 0.6 µM). (**C**) Influence of single point mutations on the antigen-binding activity of the humanized Fab version "4NRY". The chimeric αhGal3-Fab (black; K_D_ = 1.2 ± 0.1 nM), the initial CDR-grafted version "4NRY" (dark blue; K_D_ = 2.3 ± 0.2 nM), the single point mutant "4NRY"(L4M) (red; K_D_ = 1.5 ± 0.1 nM) and the single point mutant "4NRY"(E46K) (light blue; K_D_ = 1.3 ± 0.1 nM). Ovalbumin (grey) served as a negative control antigen in all three experiments. Graphics were prepared using Origin(Pro) version 2017 (https://www.originlab.com).

The coding regions of the modified light and heavy chain variable domains for each of the four acceptor Fv portions, with the grafted CDRs from M3/38 (Fig. 3), were obtained by gene synthesis including appropriate restriction sites and cloned on a derivative of the bacterial expression vector pASK88^33,34^ carrying coding regions for the human constant domains and a chloramphenicol resistance gene. The resulting recombinant Fab fragments were produced via functional secretion in *E. coli* and purified according to published procedures^9^ by IMAC from the periplasmic extract via the His_6_-tag attached to the heavy chain. Following SEC, a yield of approximately 0.5 mg soluble protein per 2 L culture was obtained for the constructs "3KYM", "4KQ3" and "4NRY". Interestingly, the version "5I8C" showed a significantly lower yield (approximately 0.05 mg). The purified Fabs were tested for binding of recombinant hGal3 by ELISA (Fig. 5), indicating a K_D_ value of 3.6 ± 0.1 nM for "4NRY", 5.9 ± 1.9 nM for "4KQ3", 6.9 ± 1.3 nM for "5I8C" but a much lower affinity for "3KYM", with K_D_ = 2.8 ± 0.6 µM. Thus, the best humanized αhGal3-Fab version, "4NRY", reached an affinity within threefold of the chimeric αhGal3-Fab (K_D_ = 1.3 ± 0.2 nM) when measured under the same conditions.

Consequently, among the four CDR-grafted constructs, "4NRY" appeared as the most promising humanized version and was subjected to refinement. In an attempt to reconstitute the full binding activity of the chimeric αhGal3-Fab, additional point mutations within the vernier zone were devised by rational analysis (see Figs. 3, 4). As result, back-mutation of the residues Lys(H46) and Met(L4) to the original rat V-sequences showed positive effects on the antigen affinity. Quantitative ELISA measurements revealed K_D_ values of 1.3 ± 0.1 nM for the Lys(H46) mutant and of 1.5 ± 0.1 nM for the Met(L4) mutant, compared with a value of 2.3 ± 0.2 nM measured for "4NRY" side by side, thus approaching the affinity of the chimeric αhGal3-Fab towards hGal3, with K_D_ = 1.2 ± 0.2 nM in this experiment (Fig. 5C). Both residues are located in close proximity to the antigen-binding site, suggesting a direct interaction with the presumed full length hGal3 antigen, which is more extended than the minimal epitope peptide visible in the crystal structure with the chimeric αhGal3-Fab.

**Figure 6:**
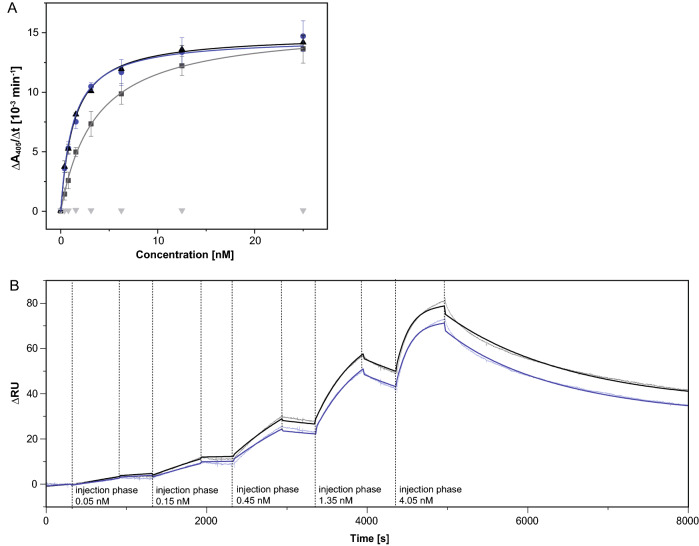
Quantification of the antigen-binding activity of GaletuzuFab. (**A**) ELISA with the recombinant hGal3 antigen immobilized on a microtiter plate. Dilution series of the chimeric αhGal3-Fab (black; K_D_ = 1.3 ± 0.2 nM), the initial CDR-grafted version "4NRY" (grey; K_D_ = 3.6 ± 0.1 nM) and the final humanized version, GaletuzuFab (blue; K_D_ = 1.3 ± 0.2 nM) were applied. Ovalbumin (grey triangles) served as a negative control antigen. (**B**) SPR analysis of the immobilized chimeric αhGal3-Fab (black; K_D_ = 0.29 ± 0.02 nM) and GaletuzuFab (blue; K_D_ = 0.34 ± 0.02 nM) using hGal3 as analyte at increasing concentrations as indicated (single cycle kinetics). Measured signals are shown in light shades while the curve fits according to a 1:1 binding model are depicted in darker colors. Graphics were prepared using Origin(Pro) version 2017 (https://www.originlab.com).

Aiming at a synergistic effect, both mutations Lys(H46) and Met(L4) were combined on the "4NRY" background, which resulted in the final humanized version, dubbed GaletuzuFab. Measured side by side with the chimeric αhGal3-Fab (GaletuxiFab), GaletuzuFab showed an indistinguishable K_D_ value of 1.3 ± 0.2 nM in the ELISA (Fig. 6). Even lower matching values of 0.34 ± 0.02 nM for GaletuzuFab and of 0.29 ± 0.02 nM for GaletuxiFab were measured in SPR experiments, which further confirmed the functional humanization of the αhGal3-Fab. Taken together, nine back-mutations to the rodent sequence were introduced into the human acceptor framework "4NRY": Ile(H2), Lys(H46), Leu(H71), Val(L2), Val(L3), Met(L4), Ile (L36), Gly(L46) and Glu(L68) (see Fig. 3).

### Site-specific labelling of the humanized Fab

Our previous PET imaging study using GaletuxiFab—in line with studies of some other tumor-targeting Fabs^10^—demonstrated the positive effect of PASylation^35,36^ to moderately increase the plasma half-life and thereby improve tumor-to-blood ratio for TC imaging in an orthotopic mouse xenograft model^9^. Therefore, the same approach was followed for GaletuzuFab and a genetically encoded, structurally disordered 200-residue sequence comprising the small l-amino acids Pro, Ala and Ser (PAS) was appended to the C-terminus of its light chain. Apart from the enlarged hydrodynamic molecular volume, similar to PEGylation^35^, the PAS sequence was employed here as a linker to expose a single free Cys residue at its other end, which subsequently was utilized for the selective chemical coupling with fluorescent or radioactive probes.

The PAS fusion protein was produced in *E. coli* and purified in the same manner as GaletuzuFab above, yielding a similar amount of 0.5 mg soluble purified protein per 2 L bacterial shake flask culture. ESI–MS analysis (Figure S1) revealed a mass of 65,185.01 Da, which is 178 Da higher than the theoretical mass of 65,007.07 Da, presumably resulting from a disulfide-bridged adduct with a *N*-acetylcysteine-methylester (177 Da) from the culture medium or the host cell metabolism. Reconstitution of the free C-terminal Cys residue was achieved via mild reduction with 1,4-dithiothreitol (DTT), followed by separation from the reducing agent by SEC^37^.

The resulting GaletuzuFab-PAS200-Cys was individually conjugated with the dye Cy7 and the chelator deferoxamine (Dfo) and analysed by ESI–MS, revealing masses of 65,838.37 Da and 65,719.44 Da, respectively, almost precisely as expected (calculated numbers: 65837.17 Da and 65,718.87 Da; see Figure S1). Further to the specific labelling of the light chain, the purified and conjugated GaletuzuFab-PAS200-Cys showed quantitative disulfide bridge formation between light and heavy chains in the same manner as the unfused GaletuzuFab, as demonstrated by SDS-PAGE (Figure S1D). In addition, ELISA and SPR measurements showed that the antigen affinity of the conjugated and PASylated GaletuzuFab was fully retained (Figure S2). Finally, the antigen-binding activity and specificity of GaletuzuFab-PAS200-Cys was confirmed by immuno-histochemical staining of human tissue sections representative for different TCs in comparison with normal thyroid tissue (Figure S3).

### GaletuzuFab radiotracer preparation and characterization

The Dfo conjugate of GaletuzuFab-PAS200 was labelled with ^89^Zr(IV) under mild conditions, followed by gel filtration on a PD10 column. The resulting specific activity was 30 ± 3 GBq µmol^−1^, and the radiochemical purity, measured via radio-TLC and SE-radio-HPLC, was > 97%. SDS-PAGE of unlabeled and labeled GaletuzuFab showed a Coomassie-stained band coinciding with the autoradiography signal, thus confirming the biochemical integrity of the radiotracer (Figure S4). The GaletuzuFab radiotracer was stable both in 0.25 M Na-acetate, 0.5 g l^−1^ gentisic acid (formulation buffer) and in human serum for up to 96 h (stable fraction > 95%). In contrast, almost complete trans-chelation of radioactivity occurred after 24 h incubation in the presence of a 1000-fold concentration of EDTA (Figure S4B). In vitro binding tests performed on FRO82-1 TC cells (RRID: CVCL_6287) revealed a K_D_ value of 15 ± 7 nM and an immunoreactive fraction of 73 ± 5% (Figure S4C).

### PET/CT imaging of orthotopic tumors using GaletuzuFab-PAS200-Dfo-^89^Zr

**Figure 7:**
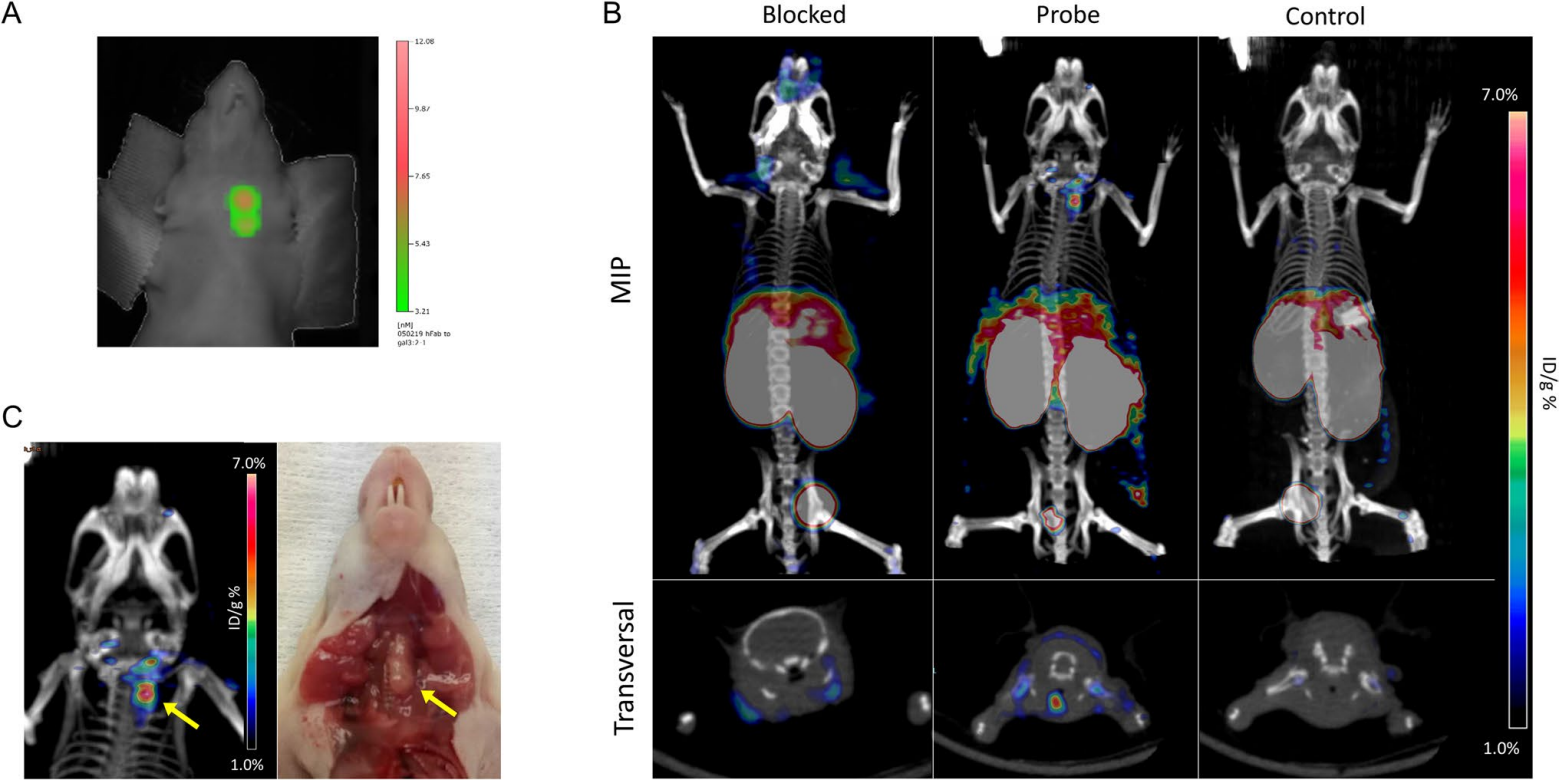
Imaging of thyroid orthotopic tumors growing in a xenograft mouse model using fluorescence-labeled and radiolabeled GaletuzuFab-PAS200. (**A**) Representative FMT imaging of a mouse bearing a FRO82-1 xenograft tumor. The scan performed 24 h after the injection of 130 µg GaletuzuFab-PAS200-Cy7 shows a hGal3-positive lesion in the neck. (**B**) PET/CT images acquired 24 h after intravenous injection of GaletuzuFab-PAS200-Dfo-^89^Zr (3 MBq) in animals from different groups: (1) co-injected with a 1000-fold excess of non-radioactive GaletuzuFab-PAS200-Cys to confirm the specificity of tracer binding in vivo (blocking experiment, left); (2) injected with the radioactive probe only (center); (3) healthy animals (without tumor) injected with the radioactive probe (control, right). The lower transversal images taken at the level of the neck area reveal the position of the tumor on the left side of the trachea while the upper images show maximum imaging projection (MIP) whole-body PET images. (**C**) Representative PET image of the neck area of an animal from the center group; the position of the PET signal corresponds to the findings at necropsy. Graphics were prepared using TrueQuant Imaging Software version 2.0 (https://www.perkinelmer.com) for the fluorescence scans and Inveon Research Workplace version 4.0 (https://inveon-research-workplace.software.informer.com) for the PET/CT image analysis.

After orthotopic FRO82-1 tumor implantation, mice were monitored weekly by visualising the lesions as hypoechoic areas via US scanning. Expression of hGal3 in the malignant nodules was confirmed by fluorescence molecular tomography (FMT) imaging of the neck region 24 h post injection of 130 µg GaletuzuFab-PAS200-Cy7 (Fig. 7). Following injection of ~ 80 µCi (3 MBq) of the GaletuzuFab radiotracer, PET/CT images were recorded at different time points (6, 12 and 24 h), revealing the best tumor-to-background ratio 24 h post injection. The radiotracer accumulation seen in the PET images correlated well with the dimensions and positions of the tumors found at necropsy (Figure S5).

GaletuzuFab-PAS200-Dfo-^89^Zr showed specific uptake in the left thyroid lobe harboring the tumor, thus allowing the imaging of malignant tissue with high contrast (Fig. 7B). PET images of a group of control animals with healthy thyroid did not reveal any signal in the neck area. Blocking experiments performed by co-injecting ~ 80 µCi of the radiotracer together with a 1000-fold concentration of unlabeled GaletuzuFab-PAS200 showed strong decrease in the signal due to the saturation of binding sites, hence confirming the target specificity of the radiotracer (Fig. 7C). Similar to the previously investigated chimeric αhGal3-Fab^9^, radioactivity also accumulated in the kidneys and liver, which is consistent with the known function of these organs in tracer metabolism and excretion, respectively^38^.

### Quantitative analysis of radiotracer accumulation

The accumulation of GaletuzuFab-PAS200-Dfo-^89^Zr in orthotopic FRO82-1 tumors calculated via image-derived analysis was 3.8 ± 0.9%ID g^−1^. Biodistribution analysis of the mice showed similar results, with an uptake of 4.1 ± 0.7%ID g^−1^ in the left thyroid lobe bearing the xenograft tumor (Figure S6). This value was around three times higher than the background value measured for the healthy right thyroid lobe (1.6 ± 0.1%ID g^−1^) which served as an internal control. The blocking experiments showed a significant decrease in signal (p < 0.05), comparable with that of the tumor-free thyroid lobe (2.0 ± 0.3%ID g^−1^). Elevated accumulation of the tracer was measured in liver (4.6 ± 0.5%ID g^−1^), spleen (11 ± 4%ID g^−1^) and kidneys (85 ± 5%ID g^−1^), i.e. the main organs for metabolisation of the radiotracer, radiometal accumulation and excretion, respectively. In conclusion, the specific accumulation in thyroid tumor and the tissue distribution measured for GaletuzuFab-PAS200-Dfo-^89^Zr matched the high contrast tumor images observed by PET. These functional features of GaletuzuFab-PAS200-Dfo-^89^Zr were essentially indistinguishable from those of the PASylated chimeric αhGal3-Fab tracer that was extensively investigated before^9^, thus providing proof of success of our antibody humanization endeavor.

## Discussion

Despite progress during the last years in improving the preoperative diagnosis of TC as well as the management of cancer patients, the identification of thyroid carcinoma remains a challenging process that is dominated by largely outdated methods and involves unnecessary surgery as a prevailing therapeutic approach^6,7^. Hence, there is an unmet need for a reliable, non-invasive procedure to identify malignant nodules, which can be distinguished only with difficulty from the highly prevalent benign thyroid proliferations (in particular nodular hyperplasia) using the currently available diagnostic tools. This is particularly evident for follicular carcinoma, follicular variants of papillary thyroid carcinoma and for carcinomas that have lost the capacity to accumulate radioiodine due to reduction or lack of the sodium iodide symporter (NIS), which therefore leads to negative or equivocal ^131^I whole-body imaging^39^. Better methods for TC diagnosis would also be important in the case of poorly differentiated (PDTC) and anaplastic thyroid (ATC) carcinomas, which are aggressive tumor types with high metastatic potential. Again, these very rare forms of thyroid carcinoma are characterized by the lack of ability to accumulate radioiodine due to the absence of NIS and, therefore, are difficult to be diagnosed and cured^40^.

In recent studies, we demonstrated that immuno-PET imaging by targeting hGal3 can be a powerful diagnostic method for TC, with high potential to aid the discrimination between malignant and benign nodules^9,41,42^. To enable the translation of hGal3 imaging into clinical application, we now have developed a humanized Fab fragment derived from the well validated hybridoma mAb M3/38^8^ that shows high affinity and specificity towards this human TC-associated antigen. In a preceding study, immuno-PET imaging experiments with a chimeric Fab of the αhGal3 mAb using an orthotopic TC mouse model^9^ revealed advantages for in vivo imaging due to the faster clearance and higher specific tumor uptake of the PASylated recombinant Fab in comparison with the full size mAb. To further lower the risk of an ADA response upon repeated administration to human patients, we have fully humanized the rat variable domains present in the chimeric Fab by CDR grafting onto human framework regions.

Whereas the concept of CDR grafting between Igs from different species was originally devised on the basis of structural considerations^13,14^, common humanization procedures mostly rely on a homology assessment at the level of the amino acid sequence till today^16,43^. This is somewhat surprising particularly in cases where three-dimensional structural information for the donor antibody actually is available^44–46^. In the early endeavors, acceptor framework sequences from structurally characterized antibodies or Bence Jones proteins were chosen^13,14,47^; this was followed by the use of human consensus Ig sequences^20^, including the famous example of the αHER2 antibody trastuzumab^15^. Today, however, frequently expressed germline Ig sequences from the human genome serve as preferred templates with presumably low immunogenicity^43,46,48^.

On the other hand, it is well known that the simple replacement of CDRs in the amino acid sequence of the acceptor Ig by those from the mAb with desired specificity can lead to incomplete preservation of the antigen-binding activity as a consequence of structural incompatibilities between the donor CDRs and acceptor frameworks at a finer granular level. Hence, the reconstitution of the original antigen affinity usually requires back-mutation of certain positions in the acceptor frameworks to the corresponding donor residues^14,17,28,48^. Such structural incompatibilities can arise from (1) altered packing between CDRs and framework regions in the donor antibody relative to the situation after grafting on the acceptor frameworks, in particular involving residues of the vernier zone^28^, (2) differences in the V_H_/V_L_ pairing—i.e. the mutual orientation of the V_H_ and V_L_ domains^43^—and (3) differences in key framework residues responsible for the canonical conformations of CDRs^31^.

To address these potential problems and identify critical residues for CDR function, structural modelling of the donor antibody was proposed soon after inception of mAb humanization by CDR grafting^17^. Nevertheless, published examples of such antibody humanization guided by rational protein design have remained scarce. One case is the successful humanization of the anti-CEA antibody T84.66 based on crystal structure data^49^; however, in this example framework regions of the αHER2 antibody 4D5 ver. 8 were chosen as acceptor, which itself is a humanized mAb based on consensus Ig sequences as explained above and not a natural human antibody.

Today, the situation with regard to the knowledge base has changed: functional fragments (mainly Fab) of several hundred human mAbs have been structurally elucidated by X-ray crystallography and are easily accessible from the Protein Data Bank at RCSB (PDB) or from specialized databases such as the AbDb^22^. Under the plausible assumption that the likelihood of success of CDR grafting rises with the degree of structural similarity between the rodent donor and the human acceptor frameworks that support the set of CDRs, we have exploited a strategy solely based on 3D-structural information. In this concept the choice of a functionally suitable natural human acceptor framework requires the precise knowledge of the structure of the rodent mAb of interest considering that modeling approaches, even though useful in the early days of this field^17^, are associated with substantial uncertainties. In the present case, the co-crystallization and X-ray structural analysis of the chimeric αhGal3-Fab with its epitope peptide provided access to that kind of information. Subsequent search in a structural antibody database led to the identification of four promising Fv regions (PDB IDs: 3KYM, 4KQ3, 5I8C, 4NRY), which were subsequently employed for experimental CDR grafting.

Not unexpectedly, the different humanized versions resulting from the initial design showed decreased antigen affinity to varying extent, ranging from 2800 nM for "3KYM", over much better values of 6.9 nM for "5I8C" and 5.9 nM for "4KQ3", to 3.6 nM for the most promising candidate, "4NRY". Remarkably, the latter design, which made use of the framework regions of the human mAb m66^27^ for CDR grafting—including some obvious back-mutations to the rat sequence—showed an affinity already within a factor 3 of the original chimeric Fab (1.3 nM). Of note, the mAb m66, which was cloned using a phage-display Fab library prepared from frozen PBMC mRNA of a patient whose serum contained cross-reactive HIV-1-neutralizing antibodies, is particularly well characterized. Its light and heavy chain sequences closely match the predicted human germ line precursors IGHV5-51*01/IGHD3-10*01/J6*02 and IGKV1-39*01/IGKJ3*01, respectively^27,50^, thus providing a reliable basis for low immunogenic potential of these acceptor framework regions.

In general, the modest loss in antigen affinity for 3 of the 4 humanization attempts validates our structural design concept of searching the most closely related light/heavy chain framework with a fixed V_H_/V_L_ pairing—compared with the strategy of choosing the most similar light chain and heavy chain frameworks individually, as it is still common practice in antibody humanization^43^. Since the likelihood of finding a most similar human V_H_/V_L_ pair for a given mAb of animal origin correlates with the size of the growing database of fully human mAb structures, this approach will become increasingly successful for future CDR grafting campaigns.

Nevertheless, plain grafting of the CDRs alone cannot be expected to guarantee full retention of the antigen-binding activity of the original rodent mAb. Thus, back-mutations to the donor sequence, in particular in the vernier region, are typically introduced to account for remaining small structural deviations^15,28^. Of course, the number of such substitutions has to be kept low in order not to compromise the goal of the humanization procedure, i.e. reducing non-human sequence stretches to minimum. In total seven back-mutations were introduced during our first design for "4NRY" due to obvious structural differences between the donor and acceptor frameworks.

At the second stage of humanization, after characterization of the initial "4NRY" version as the most promising candidate, additional back-mutations were evaluated in order to further reduce the difference in affinity compared with the original chimeric Fab. In particular, two positions attracted our attention: H46 and L4 (see Fig. 4). Residue H46 and its neighbors, H47-H49, create a platform that structurally supports CDR-H2^29^. Thereby, the side chain of H46 is located at close distance (2.5 Å) to H62 and H63, which form the tip of the CDR-H2 hairpin loop, and can influence their positions. On the other hand, residue L4 is part of the β-sheet underneath CDR-L3, in proximity to its C-terminal residue L97, and plays a role for the conformation of this hypervariable loop^29^.

The combination of both mutations eventually led to recovery of the full binding affinity, which might be explained by a more extended interaction between the complete hGal3 antigen and the Fab fragment than it appeared from the crystal structure with the truncated 9mer epitope peptide. This epitope is located in the ND of hGal3, which is described as a random coil^51^. Thus, additional contacts of the Fab with the highly flexible ND as part of the full size antigen are possible. As the two residues H46 and L4 are both located within the same groove at the V_L_/V_H_ interface, and both have sufficient accessible surface to engage in intermolecular interactions, a synergistic effect seems conceivable. As result, GaletuzuFab, which was developed in just two steps and carries only nine rat residues in the human framework regions for both Ig chains, shows an antigen affinity that is indistinguishable from the original chimeric Fab. This demonstrates the efficiency of our structure-based antibody humanization approach.

Apart from the minimization of potentially immunogenic sequence epitopes, a high degree of molecular definition of a biopharmaceutical is critical, whereas molecular heterogeneity can have detrimental effects on pharmacokinetics, in vivo efficacy and tolerability^52,53^. In this context the posttranslational modification made by introduction of the chelator Dfo for radionuclide labeling of GaletuzuFab poses a critical factor. Here, the strategy of site-specific coupling via a single free Cys residue, specifically introduced at the freely accessible C-terminus of the structurally disordered PAS tail and, thus, prone to reaction with a maleimide group^54^, was chosen. While initially incorporated to effect a moderately increased plasma half-life and boosting tumor accumulation^10^, the PAS sequence at the end of one of the Ig chains conveniently served as a spacer between the functional protein moiety and the reactive Cys residue. In this way, the need for introducing a potentially critical amino acid exchange and/or modification of the Ig scaffold, as frequently described for the construction of recombinant antibody drug conjugates (ADCs)^53^, was avoided. In fact, PASylation and labeling via the C-terminal Cys residue led to a precisely defined, monodisperse protein conjugate, as demonstrated by ESI–MS analysis, and had only marginal effect on the antigen affinity (1.9 ± 0.1 nM for GaletuzuFab-PAS200-Dfo *versus* 1.3 nM for GaletuzuFab or 1.6 nM for GaletuzuFab-PAS200-Cy7 as measured by ELISA).

Finally, the GaletuzuFab-PAS200-Dfo-^89^Zr radiotracer was prepared via labeling with the β-emitter zirconium-89 (^89^Zr; t_1/2_ = 78.41 h; E_max_ β^+^  = 0.9 meV) under mild conditions (at a temperature ≤ 37 °C) and characterized with regard to antigen-binding activity and stability. The radiochemical purity of the humanized Fab was high and similar to the one previously obtained for the chimeric αhGal3-Fab tracer (> 97%)^9^ while the specific activity was even higher (30 ± 3 GBq µmol^−1^ *versus* 19.8 ± 1.2 GBq µmol^−1^), probably due to the placement of the Dfo chelator at the sterically accessible C-terminus of the PAS chain instead of the random conjugation with Dfo isothiocyanate in the preceding study. A saturation binding assay with FRO82-1 cells indicated an EC_50_ value about one order of magnitude higher than the K_D_ measured via SPR, which can be explained by some hindered accessibility of the hGal3 antigen within the glycocalyx at the cell surface.

In PET/CT scans of mice carrying orthotopic human thyroid tumors high contrast images were obtained 24 h post tracer injection, showing a high target-to background ratio in the tumor-bearing left thyroid lobe compared to the healthy right lobe. No radiotracer uptake in the neck region of healthy animals without xenograft was visible and blocking tests with an excess of unlabeled GaletuzuFab-PAS200 further confirmed the specificity of the GaletuzuFab radiotracer, in line with biodistribution experiments. In conclusion, we were able to develop a highly homogeneous preparation of a PASylated humanized Fab tracer directed against hGal3, thus minimizing the risk of failure in a future clinical trial. The clinical potential of this novel agent for imaging and, also, therapy planning of TC has been demonstrated via high and specific uptake by orthotopic tumor xenografts in mice at early time points after injection.

## Materials and methods

### Bacterial production and purification of human Gal3

The full length human Gal3 protein (UniProtKB ID: P17931), with the unpaired thiol residue Cys173 mutated to Thr, was produced as a soluble protein in the cytoplasm of *E. coli* as previously described^9^. An N-terminally truncated fragment starting with Pro113 was prepared by amplifying the corresponding coding region with the primers 5′-GCGCATATGCCATACGGTGCTC-3′ and 5′-CGCAGTAGCGGTAAACG-3′, thus introducing an NdeI restriction site and an ATG start codon directly upstream of Pro113. The truncated P113-hGal3 protein, which carried a C-terminal *Strep*-tag II like the recombinant full-length hGal3^55^, was expressed as soluble protein in the cytoplasm of *E. coli* in 2 l shake flasks, followed by purification via Strep-Tactin affinity chromatography as well as size exclusion chromatography (SEC).

### Western blotting

Following SDS-PAGE^56^ of the recombinant full-length hGal3 and its fragment P113-hGal3, the polypeptides were electro-transferred onto a methanol-activated PVDF membrane (Merck Millipore, Billerica, MA, USA). After washing in phosphate-buffered saline (115 mM NaCl, 15 mM Na_2_HPO_4_, 4 mM KH_2_PO_4_; PBS) containing 0.01% v/v Tween 20 (PBS/T_0.01_) the membrane was incubated with the chimeric αhGal3-Fab-PAS200-Cy5.5 (2 µg ml^−1^ in PBS/T_0.01_)^9^ for 1 h at room temperature. After washing the membrane with PBS/T_0.01_, fluorescence was detected with an Odyssey fluorescence scanner (excitation: 685 nm; emission: 720 nm; LI-COR, Lincoln, NE, USA) and evaluated using Quant version 12.2 software (TotalLab, Newcastle-Upon-Tyne, UK).

### Peptide SPOT synthesis and analysis

A set of 8-mer peptides covering residues 1–112 of hGal3, with 2 residue overlap, was directly synthesized with a fully automated MultiPep RS instrument (Intavis, Cologne, Germany) according to the SPOT method^19^ on a Gly-PEG500-derivatised cellulose-membrane^57^ starting from the C-terminus using Boc-protected amino acids. After N-terminal acetylation and deprotection of the peptides, the membrane was rehydrated with PBS/T_0.01_ and incubated with the chimeric αhGal3-Fab-PAS200-Cy5.5 (2 µg ml^−1^ in PBS/T_0.01_)^9^ for 1 h at room temperature. After washing with PBS/T_0.01_, fluorescence signals were directly detected on the membrane and quantified as described for the western blot above.

### Protein crystallization

The purified chimeric αhGal3-Fab, carrying a His_6_-tag at the C-terminus of its heavy chain^9^, was dialysed against 100 mM NaCl, 10 mM HEPES/NaOH pH 7.0 and concentrated to 18 mg ml^−1^ using Amicon Ultra-30 kDa filters (Merck Millipore). This protein solution was mixed with the N-terminally acetylated synthetic peptide Gln-Ala-Pro-Pro-Gly-Ala-Tyr-Pro-Gly (Peptide Specialty Laboratories, Heidelberg, Germany) dissolved in water at a molar ratio of 1:3, and the combined solution was finally filtrated with an Amicon Ultrafree centrifugal filter (0.22 μm; Merck Millipore). After search for initial crystallization conditions using an in-house precipitant screen, crystals of the protein complex were finally grown at 20 °C using the hanging drop vapor diffusion method by mixing 1 μl of the protein/peptide solution with 1 μl precipitant/reservoir solution consisting of 25% (w/v) PEG4000, 0.1 M HEPES/NaOH pH 7.0.

### Data collection and processing, model building and refinement

A crystal was harvested, transferred into the precipitant buffer supplemented with 30% (v/v) glycerol and immediately frozen in liquid nitrogen. A single-wavelength synchroton X-ray diffraction data set at 100 K was collected at BESSY beamline 14.1^58^ (Table 1). The diffraction data were processed with MOSFLM and SCALA^59^. Molecular replacement was carried out with Phaser^59^ using the structure of the engineered Diels-Alderase Fab 1E9 (PDB ID: 3O2V)^60^ as search model. The structural model of the chimeric αhGal3-Fab was built and manually adjusted with Coot^61^. Unambiguous electron density for the peptide ligand became visible after the fourth refinement cycle and was fitted in accordance with its known sequence. Water molecules were added with ARP/wARP, rotamers of Asn and Gln side chains were adjusted with NQ-Flipper^62^, and the protein model was refined with Refmac5^59^, including model correction with the RDB_REDO server^63^. The final structural model was validated with PROVE^64^, ERRAT^65^, Verify3D^66^, PROCHECK^67^, WHAT_CHECK^68^ and the MolProbity server^69^. Secondary structure elements were assigned using DSSP^70^. Crystal contact sites were analyzed with PISA^21^ and graphics were prepared with PyMOL^71^. The atomic coordinates and structure factors of the refined chimeric M3/38 Fab·hGal3 peptide complex have been deposited at the Protein Data Bank (PDB), Research Collaboratory for Structural Bioinformatics (Rutgers University, New Brunswick, NJ, USA), under accession code 6ZVF.

### CDR grafting

As a resource for pre-numbered non-redundant crystal structures of antibody Fv portions the AbDb^22^ dataset was downloaded from http://www.abybank.org/abdb and screened for entries annotated with the species *Homo sapiens*. A Cα atom alignment with the V_H_/V_L_ moiety of the M3/38 crystal structure was performed with PDBeFold at https://www.ebi.ac.uk/msd-srv/ssm/cgi-bin/ssmserver^21^ using the following settings: chains, all chains; lowest acceptable match, 70%. Graphics were prepared with PyMOL^71^; to this end, the V_H_/V_L_ Cα coordinates were structurally aligned using the command “super” taking into account only the framework residues of both chains: L1–23, L35–49, L57–88, L98–107, H1–25, H36–49, H66–94, H103–H111^20^. This three-dimensional superposition of the αhGal3 Fv moiety with the most similar crystal structures of human mAbs from the PDB served as a basis for assessing the best structural match for CDR transplantation and for identifying crucial side chains in the rat V-regions that were then implemented into the human acceptor framework of choice (see text).

### Preparation of the vectors for *E. coli* production of the Fab variants

The V_H_ and V_L_ sequences for each human framework carrying the precisely grafted CDRs of the rat mAb M3/38 according to the definition by Kabat^20^, and amended for CDR-L1^31^, plus some back-mutated rat framework residues (see Fig. 3) were codon-optimized for protein biosynthesis in *E. coli*^72,73^ and obtained as synthetic genes from Geneart (Regensburg, Germany). The coding regions for the V_L_ and V_H_ domains were subcloned on a derivative of the bacterial expression vector pASK111^74^ which carries a Cam resistance gene and already encoded the human C_κ_ and C_H_1 domains with a His_6_ tag at the C-terminus of the heavy chain as well as N-terminal bacterial signal peptides to direct secretion of the recombinant Fab into the bacterial periplasm^33,34,75^. To this end, the V-regions were equipped with suitable flanking restriction sites: XbaI/Eco91I for V_H_ and NcoI/XhoI for V_L_. Subsequent introduction of additional back-mutations into the human framework regions was accomplished using QuikChange mutagenesis (Agilent, Santa Clara, CA, USA) of the expression plasmid pASK111-4NRY with the following primers: Lys(H46), 5′-CCTGGTAAAGGTCTGAAATGGATGGGTTGGATT-3′ and 5′-AATCCAACCCATCCATTTCAGACCTTTACCAGG-3′; Met(L4), 5′-AAAGCCGATGTTGTTATGACCCAGAGTCCGAGC-3′ and 5′-GCTCGGACTCTGGGTCATAACAACATCGGCTTT-3′. Subsequently, a synthetic gene cassette encoding 201 residues of the PAS#1 sequence^36^ was inserted at the 3′-end of the human C_κ_ encoding region using an appropriately introduced SapI restriction site following published procedures^35^. Prior to that, the codon for a free Cys residue was introduced at the C-terminus via QuikChange mutagenesis using the primers: 5′-CCGCACCGGCGGCCTGCTAAGCTTGACCTGTGA-3′ and 5′-TCACAGGTCAAGCTTAGCAGGCCGCCGGTGCGG-3′.

### *E. coli* production and purification of Fab fragments

The recombinant Fab fragments were produced in *E. coli* W3110^76^, which was grown either in shake flasks or in an 8 l bench-top fermenter. The periplasmic cell extract was prepared as previously described^33^. Purification was accomplished via immobilized metal ion affinity chromatography (IMAC) on a HisTrap HP 5 ml column (GE Healthcare, Munich, Germany) charged with Ni(II), followed by cation-exchange chromatography (CEX) on a Toyopearl Sulfate-650F column (Tosoh, Tokio, Japan) and, finally, SEC on a Superdex 75/200 26/60 column (GE Healthcare). SDS-PAGE was performed using a high-molarity Tris buffer system^56^. Protein concentrations were determined by measuring the absorbance at 280 nm using molar extinction coefficients calculated with ProtParam^77^ of 78,435 l mol^−1^ cm^−1^ for GaletuzuFab (48,306.45 Da) as well as GaletuzuFab-PAS200-Cys (65,007.7 Da). Analytical SEC was performed on a Superdex 75 h 10/300 GL column (GE Healthcare) for the plain αhGal3-Fab, and on a Superdex 200 h 10/300 GL column (GE Healthcare) for its PASylated version, using an Äkta Purifier or Explorer system (GE Healthcare) at a flow rate of 0.5 ml min^−1^ with 20 mM HEPES/NaOH, 150 mM NaCl, pH 7.5 as running buffer. ESI–MS was performed as previously described^9^ using a maXis Q-TOF instrument (Bruker Daltonics, Bremen, Germany). hGal3-binding activity was assessed by enzyme-linked immunosorbent assay (ELISA) and real-time surface plasmon resonance (SPR) analysis following published procedures^9^.

### Preparation and labelling of the Fab with a free thiol side chain

Prior to conjugation of the purified GaletuzuFab-PAS200-Cys with Dfo or Cy7 using appropriate derivatives carrying a maleimide group, the single unpaired Cys residue was liberated from mixed disulfides with other thiol compounds by mild reduction with a tenfold molar amount of dithiothreitol (DTT) in PBS at 20 °C, followed by separation of reagents and side products on a PD10 column (Sigma-Aldrich, Taufkirchen, Germany). To restore any intramolecular disulfide bonds that may have become affected by this procedure, the Fab was subsequently incubated with a 20-fold molar amount of (l)-dehydroascorbic acid (dhAA; Sigma-Aldrich) for 3 h at 20 °C. After another salt exchange on a PD10 column to 150 mM ammonium acetate (99.999% trace metals basis; Sigma-Aldrich), the Fab (2 mg/ml) was incubated with the fivefold molar amount of cyanine-7-maleimide (Lumiprobe, Cockeysville, MD, USA) or deferoxamine-maleimide (Macrocyclics, Plano, TX, USA) over night at 4 °C. Excess reagent was again removed on a PD10 column using 150 mM ammonium acetate as running buffer, and the composition of each conjugate was finally checked by ESI–MS.

### Antigen detection with the Cy7-labeled GaletuzuFab-PAS200 on tissue samples

Paraffin embedded tissue sections from papillary TC (N = 5) and non-small-cell lung-carcinoma (N = 5) were prepared as previously described^41^ and incubated with GaletuzuFab-PAS200-Cys (30 µg ml^−1^) or the HRP-conjugated version of the rat αhGal3 mAb clone M3/38 (Mabtech, Nacka Strand, Sweden)^8^ in 20 mM HEPES/NaOH, 150 mM NaCl, pH 7.5 for 1 h at room temperature. The sections were then incubated with a polyclonal rabbit anti-human light chain antibody (Dako/Agilent, Santa Clara, CA, USA) for 30 min. After washing with PBS (1.05 mM KH_2_PO_4_, 3 mM Na_2_HPO_4_, 155 mM NaCl, pH 7.4; Thermo Fisher Scientific, Waltham, MA, USA), the signal was revealed by incubation with a HRP-conjugated polyclonal mouse anti-rabbit secondary antibody for 30 min and development with 3,3′-diaminobenzidine using the Dako EnVision FLEX System (Agilent). Images were recorded using an Aperio CS2 ScanScope image capture device (Leica Microsystems, Wetzlar, Germany) and analyzed with Aperio ImageScope software version 12.3.3 (https://www.leicabiosystems.com). The human tissue sections used for this study belonged to the tissue bank of the Pathology Department of University Sapienza Sant'Andrea Hospital and were used under the allowance of the ethical committee of the Hospital. The study was carried out according to the ethical guidelines of the Helsinki Declaration under approval of the Scientific Board of Sant'Andrea Hospital (Prot. CE nr. 8391/2013).

### Labelling, functional characterization and stability test of GaletuzuFab-PAS200-Dfo-^89^Zr

The radiotracer for immuno-PET imaging was produced by labelling 130 µg of GaletuzuFab-PAS200-Dfo with 1 mCi ^89^Zr(IV)-oxalate (BV Cyclotron, PerkinElmer, Amsterdam, The Netherlands) under mild conditions as previously described^38^. The product was analysed by SDS-PAGE, radio-TLC and radio-HPLC^9,42^. GaletuzuFab-PAS200-Dfo-^89^Zr stability was evaluated at 37 °C for up to 96 h in different solutions, (1) formulation buffer, (2) human serum isolated from a healthy volunteer and (3) 50 mM (100-fold excess) DTPA (Sigma-Aldrich), using radio-TLC^9^. The following in vitro tests were performed on the anaplastic TC cell line FRO82-1 (RRID: CVCL_6287) which was cultured as previously reported^9^. The immunoreactive fraction of the radiotracer was determined using the Lindmo method^78^. The dissociation constant (K_D_) was calculated via saturation binding assay as described elsewhere^41^. All experiments were performed in triplicate.

### PET/CT imaging and tracer accumulation studies using an orthotopic tumor xenograft model

In vivo experiments were performed using an orthotopic TC xenograft model using healthy female athymic nu/nu Nude-Foxn1 mice at 6 weeks age (Charles River Laboratories, Sulzfeld, Germany) and the FRO82-1 anaplastic TC cell line^42^. Tumor growth was monitored via US scan. When the volume exceeded 65 mm^3^ the animals underwent FMT imaging of the neck region 24 h after i.v. injection of 130 µg GaletuzuFab-PAS200-Cy7^42^. Immuno-PET/CT imaging was performed at different time points, 6, 12 and 24 h after the i.v. injection of 3 MBq (~ 9 µg) GaletuzuFab-PAS200-Dfo-^89^Zr (N = 5) as previously described^9^. For control experiments, the same activity was injected into a group of healty mice (N = 5). For blocking experiments, a 1000-fold amount (~ 9 mg) of unlabeled GaletuzuFab-PAS200-Cys was co-administered (N = 5). After the PET scan, mice were sacrificed and organs were excised, weighed and measured for activity in a 2480Wizard2 γ-counter (PerkinElmer, Waltham, MA, USA) to calculate the %ID g^−1^ as previously described^42^. All experimental protocols were approved by the local authorities (Regierung von Oberbayern, Germany; License: 55.2–1-54–2532-216–15). To the extent applicable to in vivo imaging, this animal study has been reported in accordance with ARRIVE guidelines (https://arriveguidelines.org).

### Statistics

All data are presented as mean ± standard deviation (SD). Statistical analyses were performed via Prism 4.0 software (GraphPad, San Diego, CA, USA) using the Student’s t test for unpaired data. Two-sided significance level was calculated and values P < 0.05 were considered statistically significant.

## Supplementary Information

Supplementary Information
