# Supplementary material for: Effective rational humanization of a PASylated anti-galectin-3 Fab for the sensitive PET imaging of thyroid cancer in vivo
Source: Sci Rep. 2021 Apr 1;11:7358. doi: 10.1038/s41598-021-86641-0 (PMC8016950; doi:10.1038/s41598-021-86641-0)
Supplement: Supplementary file 1 — Supplementary Information [file 41598_2021_86641_MOESM1_ESM.pdf]

Supplementary Information

Effective rational humanization of a PASylated anti-galectin-3 Fab for the sensitive PET imaging of thyroid cancer *in vivo*

Emanuel Peplau[1], Francesco De Rose[2], Andreas Eichinger[1], Sybille Reder[2], Markus Mittelhäuser[2], Giorgia Scafetta[5], Markus Schwaiger[2], Wolfgang A. Weber[2], Armando Bartolazzi[4,5], Calogero D'Alessandria[2], Arne Skerra[1]*

[1]Lehrstuhl für Biologische Chemie, Technische Universität München, 85354 Freising (Weihenstephan), Germany

[2]Klinikum rechts der Isar, Nuclear Medicine Department, Technical University Munich, Ismaninger Str. 22, 81675 Munich, Germany

[4]Pathology Research Laboratory, Cancer Center Karolinska, Karolinska Hospital, SE-17176 Stockholm, Sweden

[5]Pathology Research Laboratory, Sant'Andrea Hospital, University Sapienza, via di Grottarossa 1035, 00189 Rome, Italy

*Corresponding author: Arne Skerra: email: skerra@tum.de

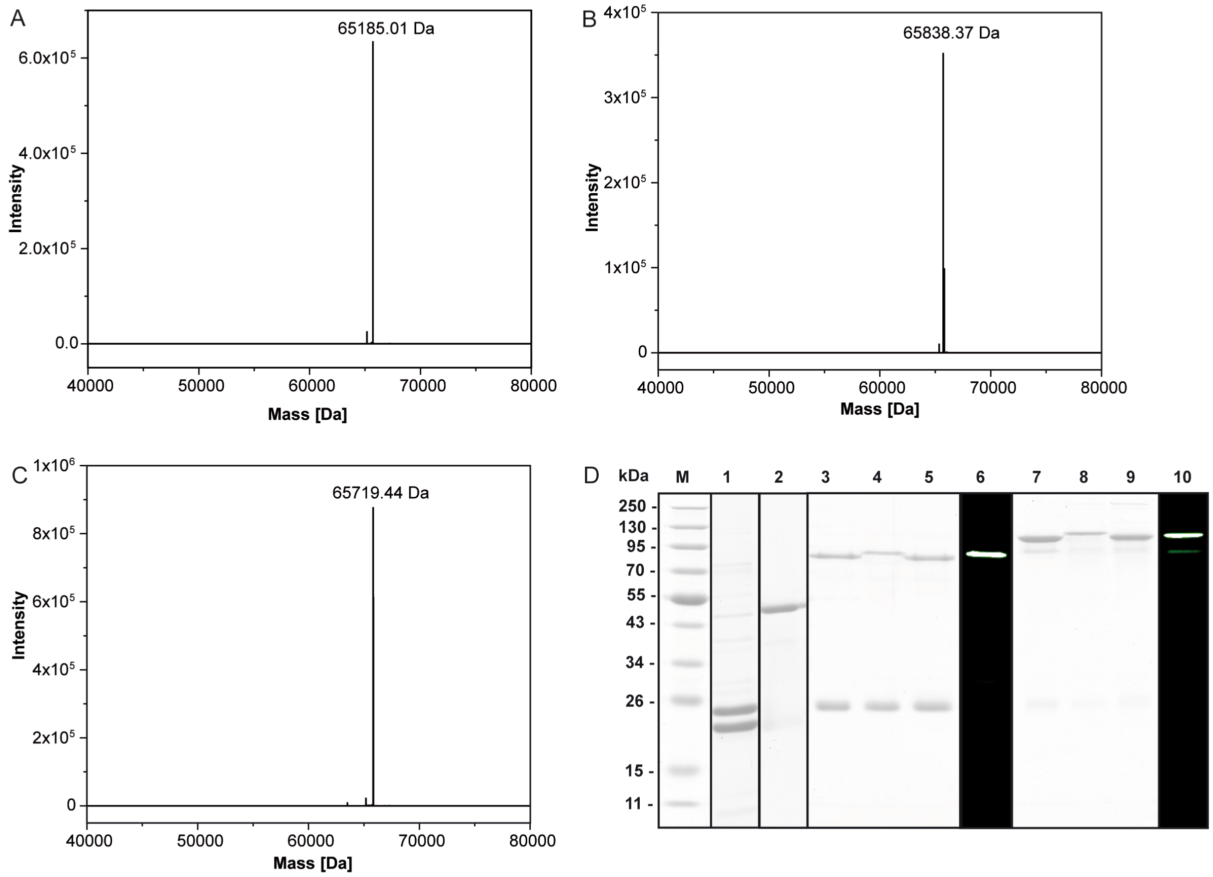

Figure S1: Biochemical analysis of GaletuzuFab-PAS200 conjugates. (A) Deconvoluted ESI-MS of the unconjugated GaletuzuFab-PAS200-Cys (calculated mass: 65007.07 Da). (B) Deconvoluted ESI-MS of the Cy7 conjugate (calculated mass: 65837.17 Da). (C) Deconvoluted ESI-MS of the Dfo conjugate (calculated mass: 65718.87 Da). (D) Coomassie Blue-stained or fluorescence-scanned (excitation: 750 nm / emission: 775 nm) SDS-PAGE of various GaletuzuFab conjugates, grouped from gels (performed under the same conditions), documenting different conjugation experiments. Lanes: M, molecular size standard; 1, GaletuzuFab; 2, GaletuzuFab (not reduced); 3, GaletuzuFab-PAS200-Cys; 4, GaletuzuFab-PAS200-Dfo; 5, GaletuzuFab-PAS200-Cy7; 6, GaletuzuFab-PAS200-Cy7 (fluorescence scan); 7, GaletuzuFab-PAS200-Cys (not reduced); 8, GaletuzuFab-PAS200-Dfo (not reduced); 9, GaletuzuFab-PAS200-Cy7 (not reduced); 10, GaletuzuFab-PAS200-Cy7 (fluorescence scan, not reduced).

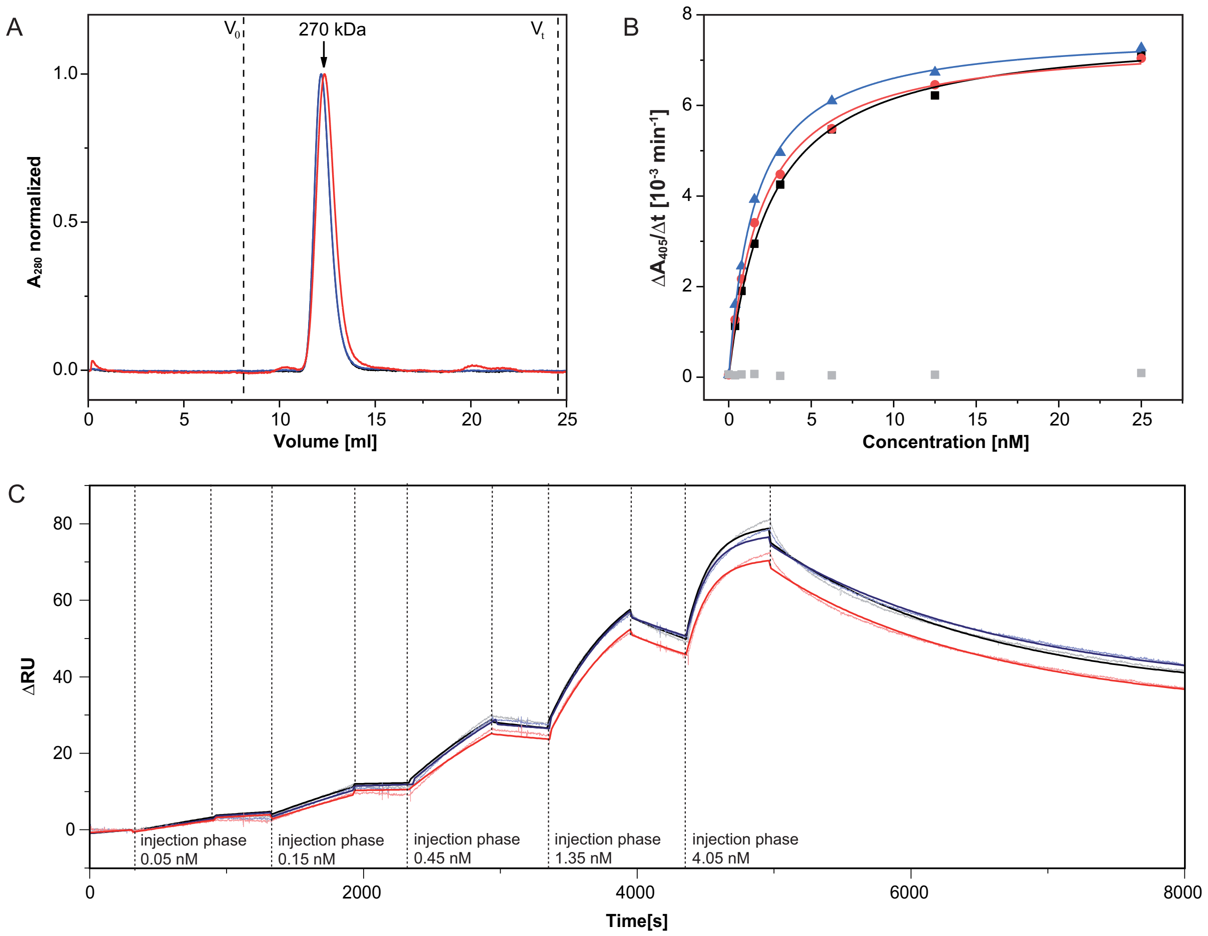

Figure S2: Biochemical characterization of the GaletuzuFab conjugates. (A) Analytical SEC of GaletuzuFab-PAS200-Cys (black), GaletuzuFab-PAS200-Cy7 (red) and GaletuzuFab-PAS200-Dfo (blue) on a S200 10/300 GL column. For all versions the apparent molecular size, as deduced from comparison with globular protein calibration standards, was 270 kDa (with just minor influence by the chemically conjugated group), thus indicating the effect of PASylation on the expanded hydrodynamic molecular volume. (B) ELISA of GaletuzuFab-PAS200-Cys (black), GaletuzuFab-PAS200-Cy7 (red) GaletuzuFab-PAS200-Dfo (blue) applied in serial dilutions against recombinant hGal3 adsorbed to the microtiter plate. The K_D values were determined by curve fitting: 1.5 ± 0.1 nM for GaletuzuFab-PAS200-Cys, 1.6 ± 0.2 nM for GaletuzuFab-PAS200-Cy7 and 1.9 ± 0.1 nM for GaletuzuFab-PAS200-Dfo. There was no cross-reactivity with BSA (light gray squares), which also served as blocking reagent. (C) SPR analysis of the interaction between recombinant hGal3, applied in rising concentrations to the GaletuzuFab-PAS200 variants which had been coupled to the carboxymethyldextran surface of the sensorchip. The K_D values were determined by single cycle kinetic fitting: 0.31 ± 0.02 nM for GaletuzuFab-PAS200-Cys, 0.24 ± 0.01 nM for GaletuzuFab-PAS200-Cy7 and 0.26 ± 0.02 nM for GaletuzuFab-PAS200-Dfo.

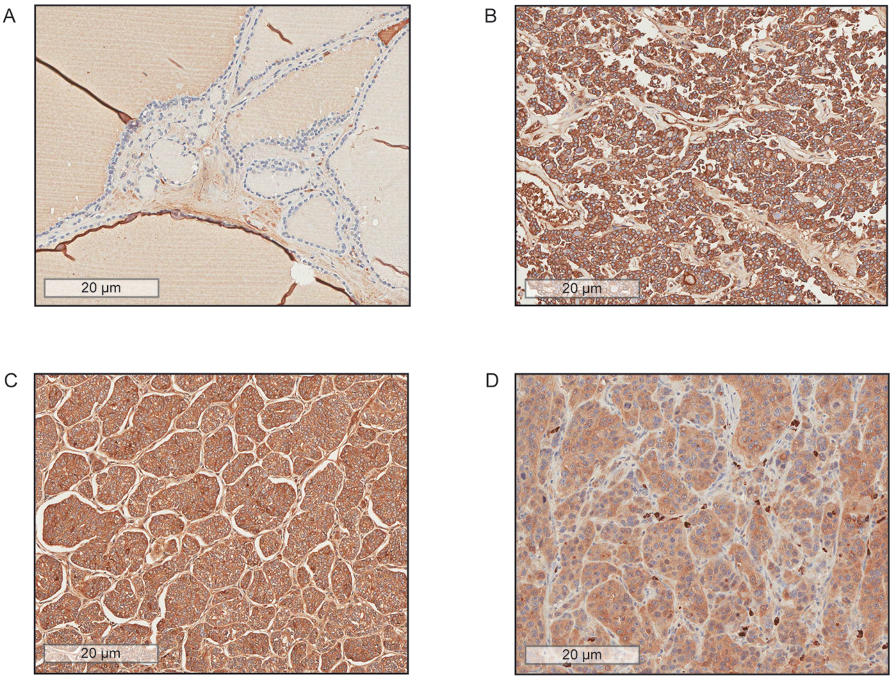

Figure S3: Antigen-binding activity of GaletuzuFab-PAS200 assessed on human tissue sections (200x magnification). A 4 µm section of the formalin/paraffin-embedded tissue was incubated with the HRP-conjugated version of the rat anti-Gal3 mAb clone M3/38 or with GaletuzuFab-PAS200-Cys (both 30 µg/ml), followed by a rabbit anti-human kappa light chain antibody and the EnVision FLEX/HRP secondary antibody. (A) No staining was visible on normal thyroid tissue with hyperplastic follicles incubated with GaletuzuFab-PAS200-Cys. (B) Strongly positive staining of hGal3 of papillary TC cells incubated with GaletuzuFab-PAS200-Cys and (C) also with the original mAb M3/38. (D) Strong staining of hGal3 expression in non-small-cell lung carcinoma with GaletuzuFab-PAS200-Cys.

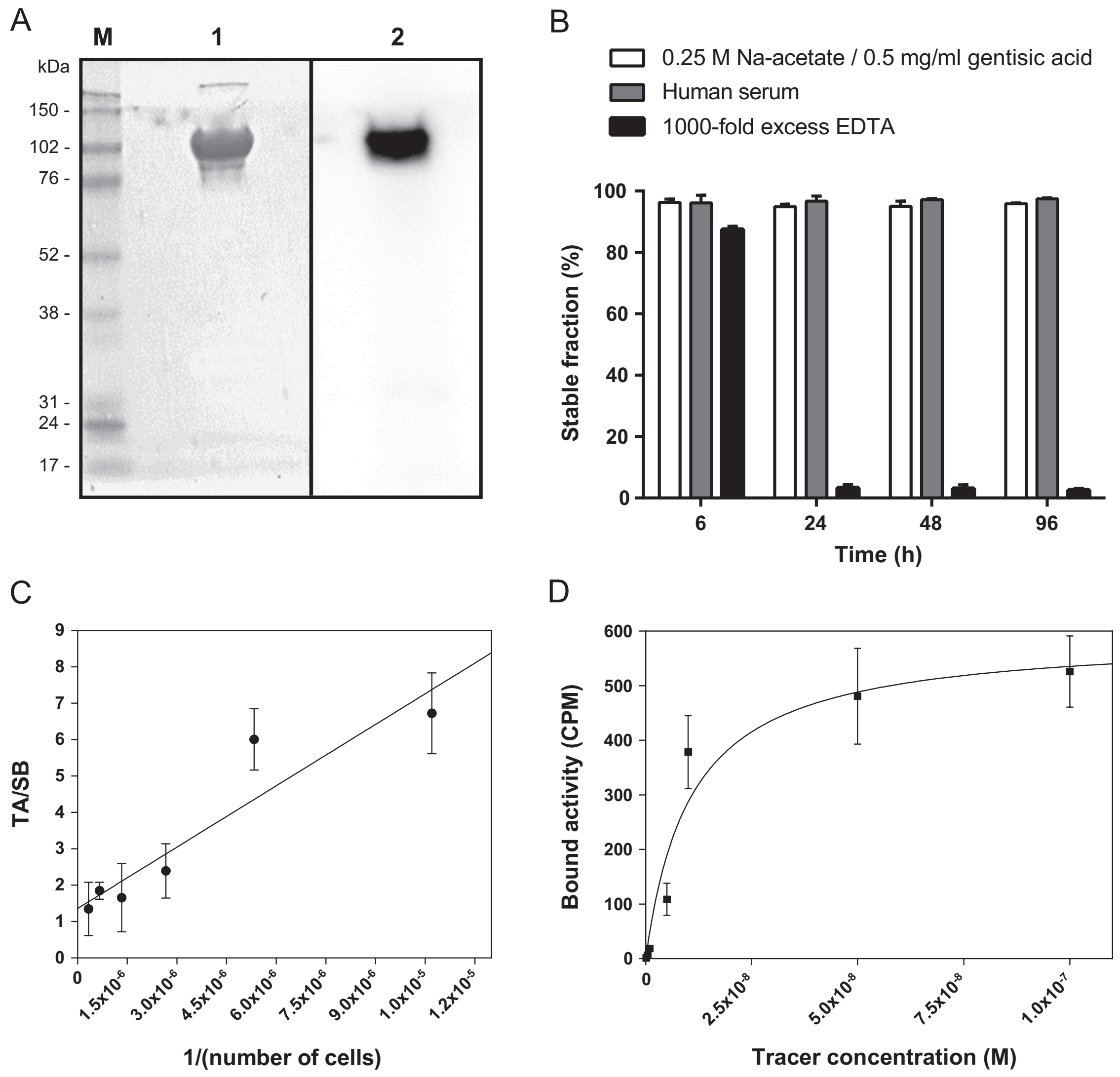

Figure S4: Characterization of the GaletuzuFab-PAS200-Dfo-^{89}Zr protein tracer. (A) The biochemical integrity of the radiotracer was assessed by SDS-PAGE (left part, lanes M and 1) under non-reducing conditions, followed by autoradiography (right part, lane 2). (B) *In vitro* stability of αGal3-Fab-PAS200-Dfo-^{89}Zr, tested in different solutions up to 96 h, was measured via radio-TLC by spotting 2 µl of the tracer solution on a silica gel-coated 60 F254 aluminium strip and eluting with 50 mM DTPA pH 4 (R_f (Fab tracer) = 0.1; R_f (free $^{89}Zr^{IV}$) = 0.8). (C) The immunoreactivity was assessed by incubating a serial dilution of FRO82-1 cells with 10 nM of GaletuzuFab-PAS200-Dfo-^{89}Zr for 2 h. The immunoreactive fraction was determined by plotting a double inverse graph of TA/SB (Total Activity/Specific Bound) *versus* 1/[normalized number of cells] and calculating the linear regression curve intercept on the ordinate axis, resulting in 73 ± 5 %. (D) Saturation binding assay using 3.0x10^{6} FRO82-1 cells/well, which were incubated with a serial dilution of GaletuzuFab-PAS200-Dfo-^{89}Zr for 2 h on ice, yielding a K_D value of 15 ± 7 nM. Data for graphs C and D were corrected for unspecific binding by subtracting the signal measured in the presence of a 1000-fold excess of unlabeled GaletuzuFab-PAS200-Dfo.

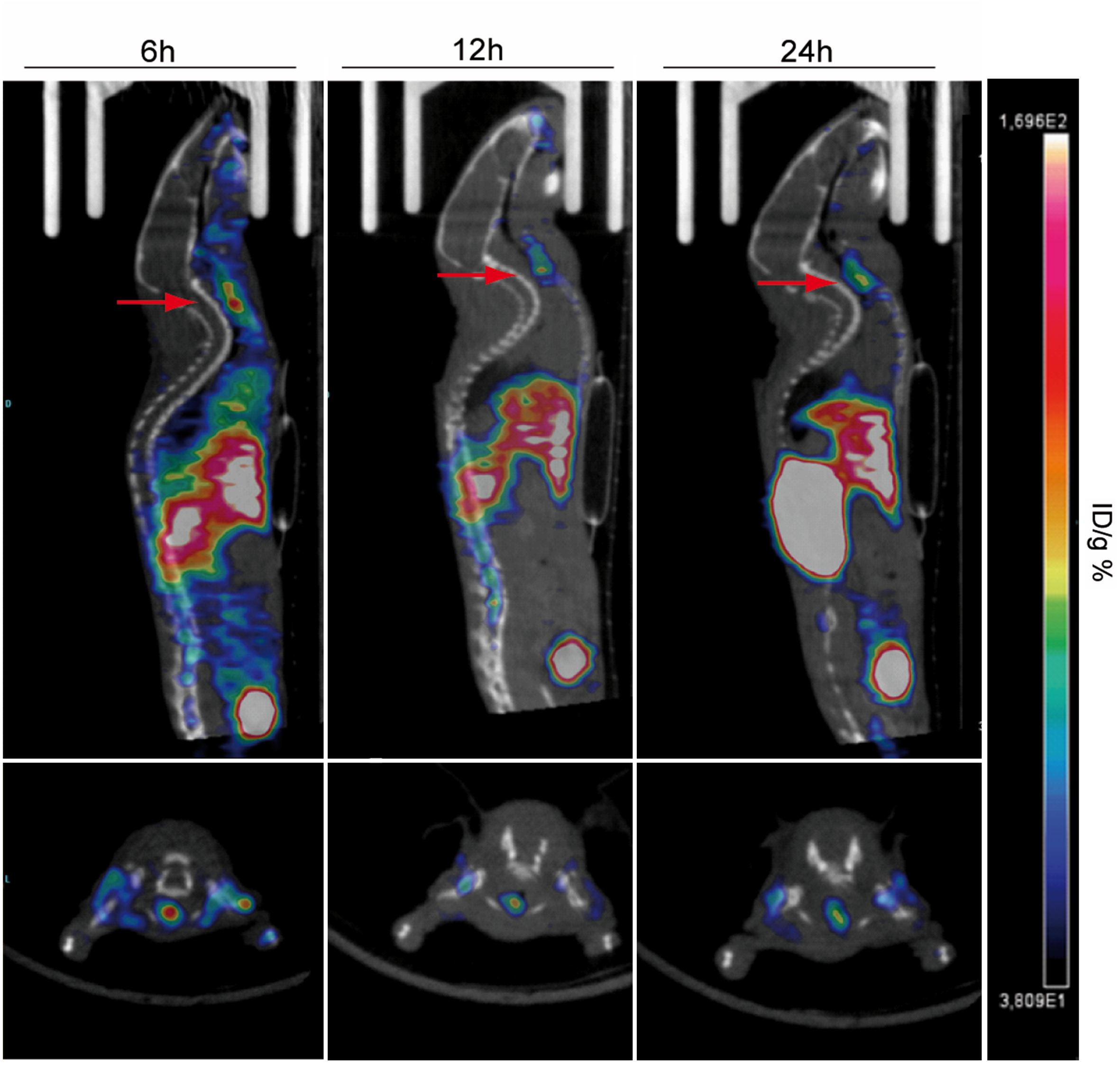

Figure S5: PET/CT images (sagittal and transversal projections) of the murine orthotopic FRO82-1 xenograft model at different time points after i.v. injection of GaletuzuFab-PAS200-Dfo-^{89}Zr (3 MBq). The best signal to background ratio was reached at 24 h p.i., allowing visualization of the tumor with high contrast.

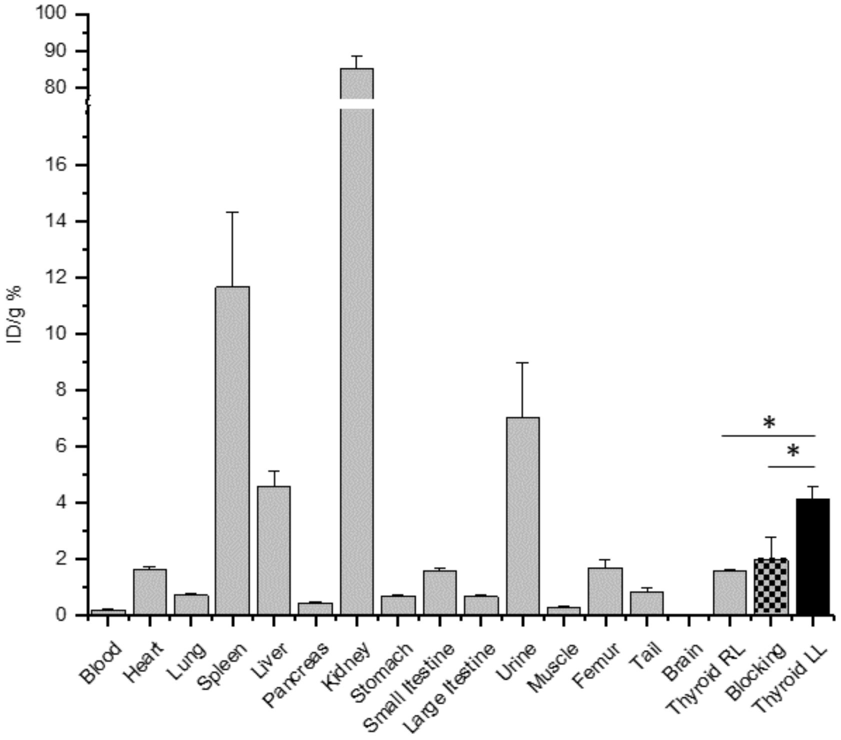

Figure S6: Biodistribution of the GaletuzuFab-PAS200-Dfo-^{89}Zr tracer in a murine orthotopic FRO82-1 tumor xenograft model 24 h p.i. Significantly elevated uptake of the tracer was found in the left thyroid lobe (LL, black bar) of one group of tumor bearing mice (N = 5) compared to the right healthy thyroid lobe used as internal control (RL). A blocking experiment was performed with a second group of tumor bearing mice (N = 5) via co-injection of a 1000-fold excess of unlabeled Fab and led to reduced uptake in the tumors (hashed bar), confirming the tracer specificity (* $p<0.05$).
